# Longitudinal analysis reveals transitions in pathogen profiles associated with mastitis in dairy cows

**DOI:** 10.1186/s13567-025-01665-y

**Published:** 2025-12-18

**Authors:** Hélène Lirot, Laurent Crespin, Patrick Gasqui, Séverine Barry, Sébastien Masseglia, Valérie Poux, Xavier Bailly, Anaïs Bompard

**Affiliations:** INRAE, VetAgro Sup, UMR EPIA, Université Clermont Auvergne, 63122 Saint-Genès-Champanelle, France

**Keywords:** Mastitis, pathobiome, clustering analysis, Markov chain models, pathogens, *Corynebacterium bovis*, *Streptococcus uberis*

## Abstract

Mastitis is a multifactorial infection of the udder potentially caused by many pathogens of varying severity and prevalence and it is one of the most common diseases on dairy farms. Limited information exists about the ecological interactions among pathogens in mastitis infections. This study aims (1) to identify the potential sources of the mastitis-causing pathogens at the farm level, (2) to analyse the statistical associations and dynamics of these pathogens over time in the milk microbiota, and (3) to assess their impact on somatic cell count (SCC) fluctuation. To address these objectives, two 4-month longitudinal studies were conducted on cows of six dairy farms in the Auvergne region of France. Milk and faeces were collected from a total of 33 cows, along with environmental samples (bedding and milk filter). A commercial qPCR kit (PathoProof^™^) was used to quantify 15 mastitis-causing pathogens in these samples. The data were then processed using Principal Component Analysis, the Ward clustering method and discrete-time Markov chain models. Clustering analyses of quarter milk samples revealed distinct profiles of pathogen distribution associated with specific SCC and cow recovery dynamics. Notably, profiles involving *Corynebacterium bovis*, though considered a minor pathogen, were associated with persistent infections and variable SCC levels. We also described co-infections between *C. bovis* and *Streptococcus uberis*. Non-*aureus* staphylococci (NAS) were associated with a wide range of effects on udder health, as they constitute a diverse bacterial group, and detection of this group in the udder might generally not be sufficient to confirm a mastitis diagnosis. We highlight the importance of considering multi-pathogen associations and longitudinal dynamics in udder health management.

**Supplementary Information:**

The online version contains supplementary material available at 10.1186/s13567-025-01665-y.

## Introduction

In dairy cattle farming, mastitis is the most common infectious disease [1]. These udder infections lead to decreased milk yield and quality, along with increased antibiotic use, all of which contribute to substantial economic losses for farmers [1, 2]. Since mastitis can become chronic and its causative pathogens can spread to other cows [3], disease management often involves early culling of affected cows [4].

Mastitis is a multifactorial disease influenced by both animal-related and environmental factors. At the individual level, factors such as genotype, lactation stage and number, udder morphology, and immune response influence the potential to develop mastitis [5]. Environmental factors related to farming practices, milking equipment and hygiene also play a role [6]. Bedding is the main source of environmental pathogens responsible for mastitis. The number and composition of bacteria in bedding vary according to the type of material used [7, 8]. In addition, one study has highlighted the potential impact of bedding type on milk bacterial composition [9].

Mastitis is categorised into two different forms: (1) clinical mastitis, identifiable by visible symptoms such as udder redness and swelling, milk clots, and systemic symptoms including fatigue and fever; and (2) subclinical mastitis, which does not manifest obvious signs of infection by a pathogen, either in terms of clinical udder presentation or in milk consistency, but which can be detected through elevated milk somatic cell counts (SCC) [10]. SCC below 200 × 10^3^ cells/mL is commonly used to define healthy udder quarters, whereas higher counts indicate mastitis [11, 12]. In this study, we use SCC > 200 × 10^3^ cells/mL as an operational definition of mastitis, indicating inflammation of the udder [12].

Somatic cells are made up of epithelial cells and leukocytes (white blood cells) usually present in milk in low quantities and their number increases in milk if an infection by a pathogen is present [12, 13]. SCC is influenced by various factors, such as lactation stage, parity, and milk production [13]. The dairy industry uses SCC to adjust the purchase price of milk from farmers [2]. Therefore, it is important to describe SCC dynamics in response to different pathogens and their impact on pathogen proliferation.

Mastitis-causing pathogens can be classified into two categories according to pathogen origin and infection pathway: environmental, originating from the farm environment, and contagious, which can spread between cows [14]. Mastitis is caused by a diverse range of pathogens of varying pathogenicity and prevalence, and many authors have distinguished major from minor pathogens [10, 15]. *Staphylococcus aureus* is one of the main causative agents in France [16]. This contagious pathogen can result in chronic infections poorly sensitive to antibiotic treatment and form biofilms, complicating control measures [17]. *S. aureus* could facilitate subsequent infections by other pathogens [18, 19]. *Streptococcus uberis*, which is considered as a less severe mastitis-causing pathogen than *S. aureus*, is increasingly detected in France [20]. This environmental pathogen can also behave as a contagious pathogen. It can be found in both healthy and clinically affected udders [21]. Alongside *S. aureus* and *S. uberis*, *Escherichia coli* is considered another major mastitis-associated pathogen [15, 22]. In contrast, several other pathogens, including *Corynebacterium bovis* and coagulase-negative staphylococci, are classified as minor pathogens that contribute to mastitis to a lesser extent [23, 24]. Apart from *S. aureus*, several bacterial species belonging to the *Staphylococcus* genus, including coagulase-negative and coagulase-positive species, are considered to be potential minor mastitis-causing pathogens, and are often grouped together as NAS (non-*aureus* staphylococci) [25].

The udder has long been considered a naturally sterile environment and thus, bacteria were generally identified as mastitis-causing pathogens once isolated during intramammary infections, when SCC is high [26]. This paradigm has persisted through the scientific literature and mastitis diagnostic procedures, although the isolated bacteria might not be the primary cause of the cow’s immune response and their identification depends on their ability to be cultured. Nonetheless, we will use this definition throughout the paper and discuss its validity.

In addition, microbial interactions may play a role in the triggering and development of mastitis. Antagonistic relationships have been reported between various mastitis-causing pathogens. Cross-inhibitory relationships have been detected in milk microbiota between *S. aureus* and other staphylococci [27]. *Staphylococcus* species secrete antimicrobial substances that can inhibit the growth of other pathogens [28]. Experimental infections have shown that *C. bovis* significantly reduces the proliferation of *S. aureus* [29]. The study of statistical co-occurrence patterns between pathogens would thus be relevant for understanding mastitis occurrence and fate. However, few studies have considered multiple mastitis-causing pathogens: most focus on one or two specific pathogens. Examination of a wide range of mastitis-causing pathogens as well as the mammary microbial diversity would enable the identification of associations that could help better understand the epidemiology of mastitis and identify new levers of control.

The teat canal, although it constitutes an anatomical barrier, also serves as a microbial reservoir to the milk cistern. Most operational taxonomic units (OTU) detected in milk have also been identified in the teat canal, suggesting circulation between the two environments and the central role of the teat canal in the colonization of the mammary gland [30]. Furthermore, the teat canal is known to host a more abundant microbial community than the milk cistern [31], making it a critical site for understanding the interplay between microbial interactions and mastitis onset.

While studies have investigated either mastitis-causing pathogens or factors influencing mastitis development, few have examined both together and have used a longitudinal framework to understand how they impact mastitis dynamics through time. The udder of cows is a complex and multifactorial environment [32], which makes it difficult to control all parameters when conducting cross-sectional studies. By contrast, a longitudinal follow-up approach facilitates comparison of samples over time for each individual cow, enabling the identification of community interaction and their interplay with discrete or continuous factors (e.g., SCC, temperature, etc.) [33, 34]. Identification of the mechanisms that influence these temporal dynamics is the key to understanding mastitis development and proposing management methods.

The objectives of this study were (1) to identify the potential sources of mastitis-causing pathogens at the farm level, (2) to analyse the associations and dynamics of these pathogens in the udder microbiota over time; and (3) to assess their impact on SCC fluctuations.

Two longitudinal follow-ups, conducted twice weekly over a 16-week period, were carried out on cows from several dairy farms in the Auvergne region of France. Milk from the teat canal and faeces from the cows and environmental samples (bedding and milk filters) were analysed using a commercial kit to detect the presence and abundance (copy number) of 15 of the putatively most common mastitis pathogens. These 15 bacterial taxa detected by the PathoProof^™^ PCR diagnostic kit will be referred to as pathogens, although not all are consistently associated with high SCC.

## Materials and methods

### Sample collection

**Figure 1:**
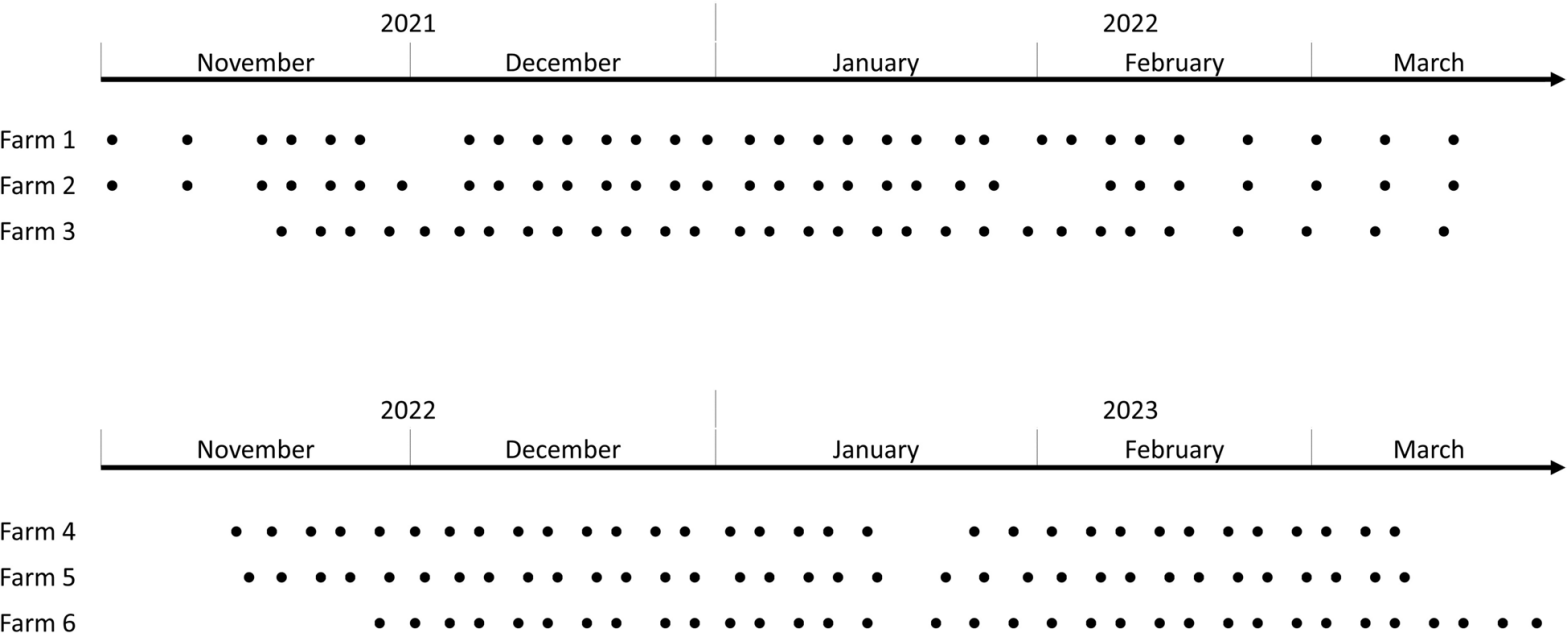
**Study design of the longitudinal monitoring. **Each dot represents a farm visit during which milk, faeces, bedding, milk filter (or bulk tank milk) samples were collected.

Two independent 16-week longitudinal sampling campaigns were conducted from November to March, the first in 2021–2022 and the second in 2022–2023 (Figure 1).

Each sampling campaign included three different commercial dairy farms in the Auvergne region of France. The farms involved in the two sampling periods were distinct. The cows were selected in collaboration with the farmers. On each farm, five multiparous lactating cows were included in the study in the first sampling period and six in the second period. To ensure longitudinal monitoring, only cows expected to remain in lactation throughout the sampling period, with no anticipated calving, were identified. Cows were then selected to obtain contrasting udder health profiles, with half exhibiting a low somatic cell count (SCC) and the other a high SCC, based on the farmer’s SCC records. The 33 selected cows belonged to three different breeds: Holstein (*n* = 21), Montbéliarde (*n* = 5) and Jersey (*n* = 7).

Milk from a single quarter and faeces samples were collected twice weekly from each cow included in the study, along with bedding and milk filter (or bulk tank milk) from the farm (Table 1 and Additional file 1). During the first follow-up, samples were collected only once per week at the beginning and end of the study period due to logistical constraints. Some sampling points were missed due to adverse weather conditions that prevented farm visits. All cows remained in the study for the entire duration, except cow 4B, who died from an injury 1 month before the end. Samples from this cow were included until her death. In addition, cow 1D was introduced 1 month after the start of sampling. Data from the culled cow were completely excluded from the analyses.

**Table 1:** **Characteristics of cows between samplings 1 and 2**

Variable	Sampling 1	Sampling 2
Farms number	3	3
Cow number	15	18
Parity	Min: 2; Max: 9; Mean: 5.2; Median: 5.5	Min: 2; Max: 7; Mean: 3.77; Median: 3.5
Breeds	5 Holstein, 5 Jersey, 5 Montbéliarde	16 Holstein, 2 Jersey

Parity values (the number of lactations per cow) are reported as minimum, maximum, mean, and median for each year. Breed composition is detailed

The same teat was used for sampling throughout the study period for each cow. Milk sampling was performed during morning milking. Approximately 4 mL of milk were manually collected from the first streams of milk of the selected teat in a sterile 5 mL tube. The combination of the farmer’s usual cleaning protocol (disinfection of the teat with a dipping solution) and a thorough additional ethanol disinfection of the targeted teat aimed to reduce contamination from the teat skin.

This specific sampling strategy, keeping the foremilk, was designed to sample the microbial populations present in the teat canal and the teat cistern. This approach deviates from the National Mastitis Council recommendations for aseptic milk sampling. Their recommendations aim to minimise contamination from the teat canal by discarding foremilk and are specifically designed to isolate mastitis-causing pathogens in a clinical diagnostic context [35], while we aimed to capture the diversity of the microbial populations colonising the udder, including the teat canal.

Signs of udder inflammation, teat swelling, or pain during sampling were recorded at each sampling. Abnormal milk appearance, such as the presence of clots, blood, or atypical coloration, was also noted, although none were observed during the entire sampling period. In addition, once a week, a second milk sample from the same teat was collected for SCC analysis.

Cow faeces were collected non-invasively, immediately after defecation, using a sterile single-use spoon and transferred into a sterile 120 mL container. The appearance and consistency of each faecal sample were recorded.

One bedding sample was collected per farm on each sampling day, using a sterile sampling sock (StériSox^®^; SodiBox, Névez, France) fitted onto a weighted boot covered with a sterile over-boot. The boot and StériSox^®^ were applied at ten points on the cow bedding area, then placed into a sterile pouch supplied with the kit.

At the end of the morning milking, the milk filter from the milking machine, through which all the herd milk had passed during that milking session, was collected by the farmer and transferred into a sterile pouch. On farm number 5, where no milk filter was available, 300 mL of bulk tank milk were sampled instead, using a disinfected measuring cup, and stored in sterile tubes.

All samples were transported to the laboratory in a cool box and stored at 4 °C within two hours of collection.

### Farm management

The farm’s general husbandry practices were recorded prior to the start of the sampling period. In addition, on each sampling day, a standardised questionnaire was administered to collect information on farm management. Farmers were asked about any changes that had occurred on the farm, such as modifications to bedding, feed, herd-level treatments, as well as the health status of the selected cows, including clinical symptoms or administered treatments. Additional background information on selected cows was also recorded, including breed, date of last calving, and parity.

### Laboratory analysis

The laboratory analyses comprised two complementary components: weekly SCC measurement in milk samples to monitor udder health, and molecular biology procedures applied to all sample matrices to quantify pathogen DNA and characterise microbial profiles.

### Somatic cell count analysis

SCC was measured weekly throughout the sampling period on a dedicated milk sample collected from the selected teat. Analyses were performed by Agrolabs (Clermont-Ferrand, France), a specialised laboratory, using a reference epifluorescence-based method [36].

### Sample preparation

Milk samples were stored at −20 °C on the day of collection until DNA extraction. Solid samples, including faeces and milk filters, were suspended in 1 mL of phosphate-buffered saline (PBS 1X) per gram of sample and homogenised using a BAGMIXER 400 S^®^ (Interscience, Saint-Nom-La-Bretèche, France) for 30 s at speed 3 (8 strokes/s) on the day of sampling. For the bedding samples, the entire StériSox^®^ was immersed in 40 mL of PBS 1X and homogenised using the same procedure. The resulting solutions were then stored at −20 °C until DNA extraction.

Bulk tank milk samples were also stored at −20 °C after collection. For each sample, four 50-mL aliquots were transferred to sterile Falcon tubes and centrifuged at 5250 *g* for 30 min at 4 °C. Supernatants were discarded, and the resulting pellets were resuspended in 1 mL PBS 1X, using the resuspension from one tube to dissolve the pellet from the next. The final combined solution was then centrifuged at 13 000 *g* for 5 min at 4 °C. The resulting pellet was resuspended in 500 µL of PBS 1X and used for DNA extraction on the same day.

### DNA extraction

DNA was extracted using the ZymoBIOMICS^™^ DNA Miniprep Kit. Each batch of 24 extractions included a negative control (PBS 1X) and a positive control.

For faeces and bedding samples, the positive control consisted of a pooled faecal sample from multiple cows and farms on the first day of sampling. For milk and filter samples, a pooled milk sample was used. Positive controls were aliquoted into single-use tubes for each extraction run and reused consistently across both sampling years.

For milk and filter samples, 250 µL of sample were added to a ZR Bashing Bead Lysis Tube, along with 750 µL of ZymoBIOMICS^™^ Lysis Solution and 20 µL of Proteinase K (20 µg/µL). After vortexing for 5 s, the mixture was incubated overnight at room temperature, then processed using a Precellys^®^ homogeniser (Bertin Technologies, Montigny-le-Bretonneux, France) at 9000 rpm for 1 min, repeated four times with a 2-min break.

For faecal and bedding samples, 150 µL of sample solution were mixed with 120 µL of PBS 1X in a ZR Bashing Bead Lysis Tube, and 750 µL of ZymoBIOMICS^™^ Lysis Solution were added subsequently. Homogenization then followed the same Precellys protocol as described for milk samples.

Lysates were centrifuged at 8000 *g* for 1 min. Four hundred µL of the supernatant were filtered using a Zymo-Spin^™^ III-F Filter and centrifuged again for 1 min at 8000 *g*. DNA purification was then carried out according to the extraction kit protocol.

To remove potential inhibitors from faecal and bedding DNA extracts, the eluate was further purified using Zymo-Spin^™^ III-HRC filter columns with 600 µL of ZymoBIOMICS^™^ HRC Prep, followed by centrifugation at 8000 *g* for 3 min. The eluate was then passed through a Zymo-Spin^™^ II-µHRC column and centrifuged at 16 000 *g* for 3 min. DNA was stored at 4 °C for short-term use and at −20 °C for long-term storage.

### DNA quantification

Absolute quantification of 16S rRNA gene copies was performed using quantitative PCR (qPCR) with the SsoAdvanced Universal SYBR^®^ Green Supermix kit (BioRad, Ref: 1725272; Hercules, California, USA) on a CFX96 Biorad thermocycler. The reaction mix consisted of 10 µL of SYBR Green Supermix, 0.75 µL of 20 µM solution of each of primers 515F (5′-GTGYCAGCMGCCGCGGTA) [37] and 928R (20 µM) (5′-CCCCGYCAATTCMTTTRAGT) [38], 3.5 µL of ultrapure water, and 5 µL of DNA template.

The qPCR program for amplifying the 16S rRNA gene included an initial denaturation step at 95 °C for 30 s, followed by 40 cycles of denaturation at 95 °C for 30 s and hybridization-elongation at 60 °C for 3 min. A melting curve analysis was performed at the end of the amplification to assess the specificity of the amplification.

### Pathogen detection

DNA extracts were analysed using the commercial PathoProof^™^ Complete-16 kit (Thermo Fisher Scientific, Waltham, Massachusetts, USA) on an Applied Biosystems^™^ QuantStudio qPCR device (Applied Biosystems, Waltham, Massachusetts, USA). This protocol enables semi-quantitative detection of the *Staphylococcus* ß-lactamase gene (conferring penicillin resistance) and the following15 mastitis-associated pathogens:

- *Corynebacterium bovis*
- *Escherichia coli*
- *Enterococcus* spp. (Including *faecalis* and *faecium*)
- *Klebsiella* spp. (Including *oxytoca* and *pneumonia*)
- *Mycoplasma bovis*
- *Mycoplasma* spp.
- *Prototheca* spp.
- *Serratia marcescens*
- *Staphylococcus aureus*
- *Staphylococcus* spp.
- *Streptococcus agalactiae*
- *Streptococcus dysgalactiae*
- *Streptococcus uberis*
- *Trueperella* (*Arcanobacterium*) *pyogenes* and/or *Peptostreptococcus indolicus* (reported jointly as *T. pyogenes/P. indolicus*)
- Yeasts

The accompanying Thermo Scientific^™^ PathoProof^™^ Norden Lab Studio^™^ software provides semi-quantitative data classified as + + + , + + , + , ± , and 0, along with the corresponding cycle threshold (CT) values.

To convert the semi-quantitative CT data into approximate pathogen copy numbers, two calibration strategies were employed. First, dilution series of the positive controls of the kit were used to generate CT ranges from 15 to 38. Second, dilution series were created from DNA extracts of two *S. aureus* and *Staphylococcus xylosus* strains (ranging from 10 genome copies/µL to 10^6^ copies/µL) to distinguish *S. aureus* and non-*aureus* staphylococci (hereafter NAS). The *Staphylococcus* spp. DNA reference material was kindly provided by Dr Sabine Leroy, who conducts research on these bacteria (joint research unit Microbiology, Digestive Environment, Health–MEDIS—University Clermont Auvergne, INRAE, Clermont-Ferrand, France.

### Statistical analysis

All analyses were performed with R version 4.2.2 using packages obtained from the Comprehensive R Archive Network (CRAN).

### Estimation of pathogen copy number from CT values

Cycle threshold values obtained from the PathoProof^™^ kit were converted into proxies for pathogen copy numbers using linear regression models calibrated on the kit’s standard dilution series and the in-house *Staphylococcus* spp. dilution series described above.

For each pathogen, the slope and intercept of the relationship between CT values and the base-10 logarithm of the genome copy number were estimated based on (i) the defined calibration range, assigning an arbitrary value of 10 copies to the lower bound, and (ii) known concentrations for *S. aureus* and *S. xylosus*. In the first case, the number of genome copies (N) was then estimated from each CT value using the following formula.

1 $$N = 10^{{\left( {\frac{CT - intercept}{{Slope}}} \right)}}$$

In the second case, to separate the data for NAS from that of *S. aureus* in the results of *Staphylococcus* spp. quantification, we assumed that $\Delta CT = CT_{SP} - CT_{SA}$, $X_{SP} = 2^{ - \Delta CT} *X_{SA}$ and $X_{SP,SA} = X_{SA}$ where *X* refers to estimated copy numbers [39]. This enabled derivation of the CT value attributable specifically to NAS when the CT value for *Staphylococcus* spp. was lower than the CT value for *S. aureus*:

2 $$CT_{SP,NA} = log \left( {2^{ - \Delta CT} - 1 } \right) *a + CT_{SA}$$

where SP refers to *Staphylococcus* spp., SA to *S. aureus*, SP,SA to *S. aureus* detected by qPCR for *Staphylococcus* spp., and SP,NA to the detection for *Staphylococcus* spp. corresponding only to NAS. The variable α represents the slope for the detection of the *S. aureus* strain, estimated from the calibration curve (see above). In cases where the CT value for *Staphylococcus* spp. was greater than or equal to the CT value for *S. aureus* (*n* = 39), we set to zero the copy number of NAS. All copy number estimates were normalised based on the sample volume used for DNA extraction: 250 µL for milk and milk filter samples, and 150 µL for faeces and bedding samples. Final estimates were expressed as genome copies per mL of the original sample.

### Somatic cell count

Quarter-level SCC values (one quarter per cow), sampled weekly, were log-transformed before linear interpolation on the SCC using the approx() function in the stat R package (method = “linear”) to produce a bi-weekly time series coherent with milk sampling for pathogen detection. Values were then back-transformed to the original scale by exponentiation. Interpolation was performed independently for each cow’s quarter, resulting in 477 values added across the dataset, corresponding to an average of 14.4 points per cow. The original dataset contained 532 data points prior to interpolation, with an average of 16 points per cow.

### Principal component analysis

A principal component analysis (PCA) was used to explore patterns in the abundance, i.e. copy numbers, of the 14 identified pathogens across four sample types: milk (*n* = 1008), milk filters (*n* = 171), faeces (*n* = 917), and bedding samples (*n* = 189). *M. bovis* was excluded from the analysis due to its absence in all samples. Pathogen copy number estimates were transformed using the natural logarithm + 1 prior to PCA, i.e. log (abundance + 1). PCA was then performed using the PCA() function with standardization (scale.unit = TRUE) from the R package FactoMineR. Visualisations were produced using the plot.PCA() function.

### Clustering milk samples

We used clustering to group samples with similar characteristics; these groups being referred to as pathogen profiles. Pathogen profiles in milk samples (*n* = 1008) were clustered based on log-transformed estimated copy numbers described above. A Euclidean distance matrix was computed using the dist() function in the R stats package (method = “euclidean”). Several criteria were used to determine the optimal number of clusters: the within-cluster sum-of-squares, silhouette index, and the gap statistic, using the fviz function from the factoextra R package (Additional file 2). Hierarchical agglomerative clustering was then performed using Ward’s method [28] implemented in the hclust() function with “ward.D2” option following [29]. The results were visualised using a multidimensional scaling representation, obtained with the cmdscale() function (stats package) (Additional file 3). Clusters were characterised by calculating the mean, median, minimum, maximum, and standard deviation of the copy number for each pathogen (Additional file 4). Robustness of the clustering analysis was assessed by re-running the clustering several times after randomly removing 10, 15 and 20% of the samples (Additional file 5).

### Markov chain modelling

A visual representation of the succession of pathogen profiles over time for each cow is provided in Additional file 6. Markov chains were used to model these transitions between pathogen profiles and characterise profile persistence over time. The persistence probability of a profile refers to the probability of staying in the corresponding state at the next time step. A probability of 1 corresponds to an absorbing profile (i.e., once entered, it cannot be left), while lower probabilities correspond to transient profiles, which tend to change more frequently. For each profile, the stationary distribution was obtained to characterise the long-term behaviour of the Markov model. The stationary distribution, or steady-state distribution, describes the long-term equilibrium of the system. It illustrates the expected proportion of time spent in each state at the Markov chain equilibrium.

Profiles identified by clustering were treated as discrete Markov Chain states, applied to both initial cluster-defined pathogen profiles or more stringent homogeneous infectious states (Additional file 7). The “more stringent” classification used more stringent assignation criteria based on rules on the presence or absence of key structuring pathogens. Transition matrices were similar whether based on initial profiles or on the more stringent homogeneous states. Results were similar for both classification (see Additional file 7 for definition of state and Additional file 8 for clustering comparison). Transition matrices between these different states were fitted using the markovchainFit() function from the markovchain R package (method = “mle”) [40] (Additional file 8). The time step of the Markov chain corresponds to the interval between samplings, which is approximately 3–4 days. NA values were inserted as needed to maintain consistent time intervals in the time series, and thus to consider irregularities from sampling gaps. Diagrams representing transition matrices were created using Python (version 3.11.0) and the pandas, network, and matplotlib libraries.

### SCC variation and profile transitions

To investigate the relationship between udder health and pathogen profile dynamics, relative SCC change rates were calculated across transition events. This was done between: (i) the transition day and the previous sampling, and (ii) the two samplings preceding the transition. The SCC change rates were calculated by dividing the difference in SCC values by the number of days between two consecutive samplings. *T*-tests were used to compare SCC trajectories, especially decreases, were linked to infection healing and transition between profiles.

## Results

### Overview of sample collection

To contextualise the analyses, a total of 2285 samples were collected during the two longitudinal campaigns. We collected 1008 milk samples, 171 milk filter and bulk milk tank samples, 189 bedding samples, and 917 faeces samples. Each of the 33 cows was sampled regularly over a 16-week period, resulting in between 23 and 32 sampling time points per cow, depending on year and specific constraints (Additional file 1). Sampling frequency was twice per week, except at the beginning and end of the 2021–2022 campaign, when it was reduced to once a week (see the “Materials and methods” section). Compared to the planned sampling, a few points were sporadically missed due to logistical constraints or extreme weather. Overall, the comprehensive design provided high-resolution, repeated measures from individual animals and farms.

### Pathogen detection across matrices

Pathogen richness varied across matrices. According to the PathoProof^™^ qPCR panel, 25% of milk samples did not show traces of pathogens, compared with only 4.5% of milk filter samples and less than 3% of bulk tank milk, bedding and faeces samples. Milk samples exhibited low pathogen diversity, with a median of 2 different pathogens per sample (Interquartile range (IQR): 1–3), whereas milk filter samples harboured higher diversity, with a median of 9 (IQR: 7–11). Bulk tank milk and faecal samples showed intermediate diversity, with medians of 6 (IQR: 5–7) and 5 (IQR: 4–6), respectively. Bedding samples were also highly diverse, with a median of 7 (IQR: 6–8) different pathogens per sample. These results indicate that environmental matrices and milk filters generally support higher pathogen diversity than individual milk samples.

**Figure 2:**
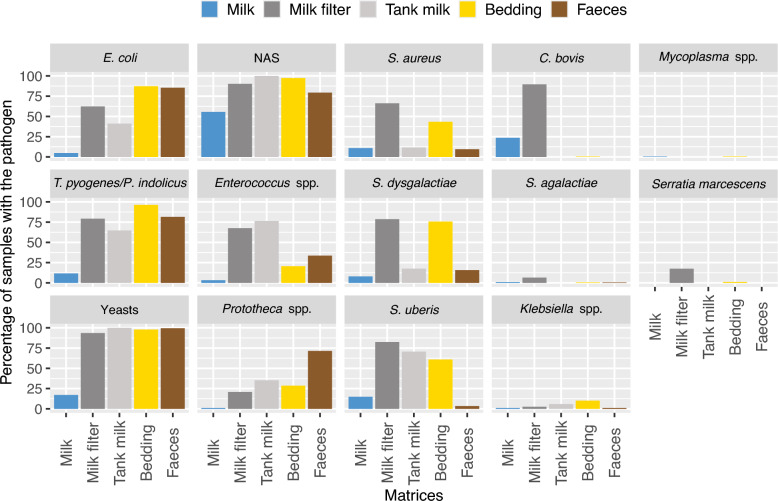
**Prevalence of mastitis-causing pathogens in different matrices:** milk (*n* = 1008), milk filter (*n* = 154), bulk tank milk (*n* = 17), bedding (*n* = 189) and faeces (*n* = 917). The colours represent the different matrices, with each plot corresponding to a specific pathogen. NAS stands for non-*aureus* staphylococci. Milk filters were used for all herds except herd 5, where bulk tank milk was sampled instead due to the absence of a filter.

The distribution of pathogens varied across matrices (Figure 2). The most frequently detected pathogens in milk samples were non-*aureus* staphylococci (NAS) (in 55.6% of samples) and *Corynebacterium bovis* (23.7%). In contrast, NAS (90.3%) and yeasts (93.5%) were predominant in milk filter samples and were consistently present in all bulk tank milk samples. In bedding samples, the most prevalent pathogens were yeasts, NAS and *Trueperella pyogenes* and/or *Peptostreptococcus indolicus*, which are detected jointly by the qPCR kit and reported thereafter as *T. pyogenes/P. indolicus* since they could not be distinguished. All of these pathogens were detected in over 95% of the samples. Similarly, faecal samples exhibited high prevalence rates for yeasts, *T. pyogenes/P. indolicus*, and *Escherichia coli* (all above 80%), while NAS was present in 79% of the samples.

*C. bovis* was specific to milk samples (23.7%) and milk filters (89.6%). It was present in only one milk sample from farm 5 and absent from the corresponding bulk tank milk samples from this farm. Other pathogens, such as *Staphylococcus aureus*, *Streptococcus dysgalactiae*, and *Streptococcus uberis*, were mainly found in milk filter and bedding samples, but rarely detected in milk and faecal samples (< 16%). In contrast, *E. coli*, *T. pyogenes/P. indolicus*, NAS, and yeasts were frequently identified in bedding and faeces (≥ 75% of samples) and also detected in over 50% of milk filter samples. No *Mycoplasma bovis* was detected throughout the study.

In addition to matrix-specific trends, between-farm variability was also evident, particularly in milk filter samples. For example, *S. aureus* was detected in only one sample from farm 1 but was found in 100% of milk filter samples from farm 4.

### Differences in pathogen abundance patterns across matrices

**Figure 3:**
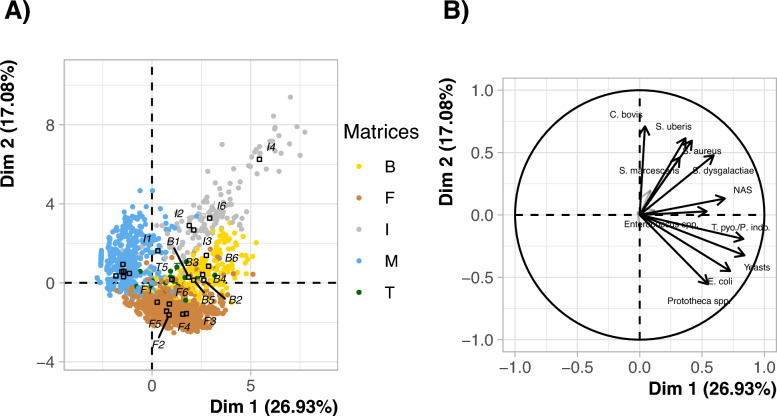
**PCA analysis of pathogen abundances among samples:** **A** Projection of samples on the first factorial plane and **B** circle of variable contributions for the first two dimensions of principal component analysis (PCA) of pathogen copy number in all samples. Only variables with a cos2 (quality of representation) greater than 0.2 on one of the first two components are displayed. The colours represent the different matrices. The labels indicate the matrix: B for bedding, F for faeces, M for milk, I for milk filters and T for bulk tank milk, followed by the farm number (1–6) (e.g., M1 designates milk samples from farm n°1). Farms 1–3 correspond to the first sampling period, while farms 4–6 correspond to the second period. Milk filters were used for all herds except herd 5, where bulk tank milk was sampled instead due to the absence of a filter. NAS = non-*aureus* staphylococci. T. pyo./P. indo. = *T. pyogenes/P. indolicus.*

Principal Component Analysis (PCA) of pathogen copy number data confirmed the matrix-specific microbial compositions observed in detection and prevalence analyses (Figure 3). The first axis primarily separated milk samples from the other matrices (bedding, faeces, and milk filters), with milk samples showing a negative correlation and other matrices being positively correlated with the axis (Figure 3A). This axis was primarily associated with the presence of yeasts (Pearson coefficient of correlation: r = 0.84, *p*-value < 0.001), *T. pyogenes*/*P. indolicus* (r = 0.83, *p*-value < 0.001), *E. coli* (r = 0.73, *p*-value < 0.001), and NAS (0.68, *p*-value < 0.001). The second axis distinguished faecal samples from other sampling matrices, with faeces characterised by a negative correlation with this axis. It was mainly structured by the presence of *C. bovis* (r = 0.71, *p*-value < 0.001) and *S. uberis* (r = 0.62, *p*-value < 0.001) (Figure 3B). These patterns highlight specific pathogen-matrix associations.

The circle of variable contributions identified different groups of pathogens (Figure 3B). *C. bovis* appeared in isolation. Next, *S. uberis, S. aureus* and *S. marcescens* clustered together, with *S. dysgalactiae* positioned nearby. NAS and *Enterococcus* spp. grouped together. *T. pyogenes/P. indolicus*, yeasts, *E. coli* and *Prototheca* spp. formed another group. *Klebsiella* spp., *Mycoplasma* spp. and *Streptococcus agalactiae* were poorly represented in the first two dimensions of the factorial plane as indicated by their cos^2^ values below 0.2. However, they played a structuring role in the following dimensions, 3 (7.44% of the total variance), 4 (7.19% of the total variance) and 5 (7.06% of the total variance) respectively.

The effect of the sampling campaign, represented by the farms (1–3 for the first campaign and 4–6 for the second session), was far less important in this analysis than the effect of the sampling matrices. The milk filter samples were very isolated, though scattered, in the first factorial plane. The bulk tank milk samples from farm 5 were located approximately at the intersection of the milk filter, bedding and faeces samples.

### Pathogen profiles in milk samples

To identify the main associations of pathogens in the milk samples, a hierarchical clustering analysis was carried out. Based on the within-cluster sum-of-squares curve, the silhouette index, the gap statistic, and considering biological relevance, we selected to partition the samples into 6 clusters for further analysis (Additional file 2). Each cluster, hereafter referred to as a “profile”, grouped samples sharing similar characteristics.

**Figure 4:**
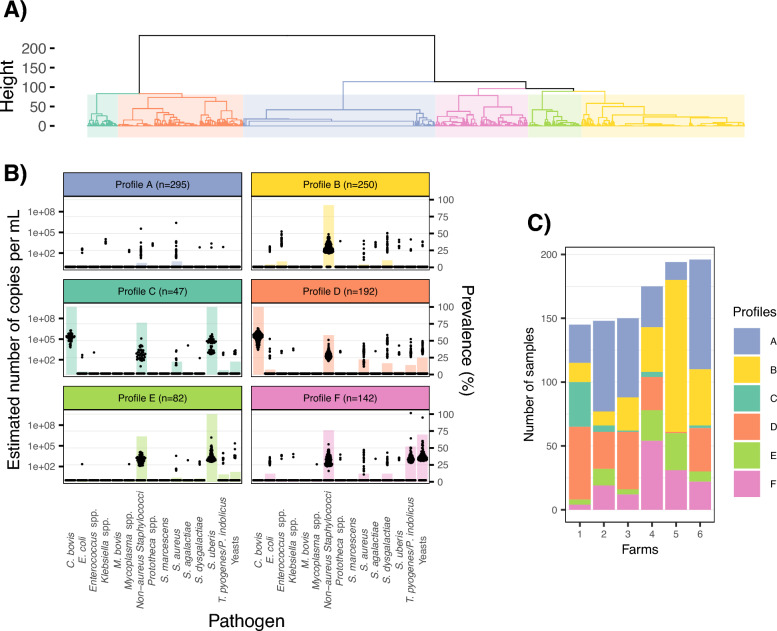
**Characterization of pathogen profiles from milk samples.** (**A**) the dendrogram shows the result of hierarchical clustering with 6 distinct profiles (**B**) distribution of pathogen copy number and prevalence by pathogen profile: the x-axis shows the individual pathogens; the left y-axis (log-transformed) represents pathogen copy number, with individual data points displayed using a quasi-random jitter to improve visualization of the distribution; the right y-axis and the bar plot represent pathogen prevalence. Results are separated by profiles identified by clustering. N corresponds to the number of samples per profile. NAS stands for non-*aureus* staphylococci. (**C**) Number of samples per farm according to the different pathogen profiles. The colours correspond to the profiles identified by clustering.

**Figure 5:**
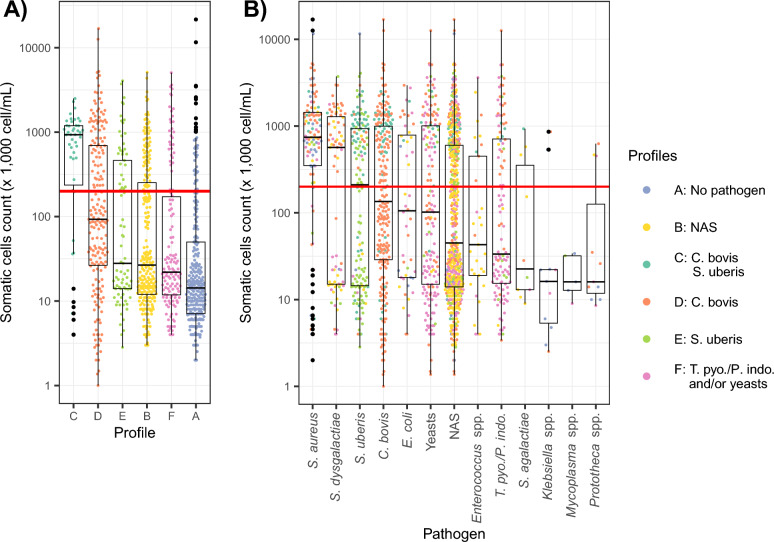
**Distribution of SCC associated with** **A** **profiles and** **B** **pathogens, ordered by median SCC**. Points are coloured according to the profiles identified through clustering, with the legend showing the key pathogens defining each profile. The data are displayed using a quasi-random jitter to improve visualization of the distribution and boxplot. The red horizontal line represents the threshold of 200 × 10^3^ cells/mL, commonly used for mastitis detection. NAS stands for non-*aureus* staphylococci.

These six profiles were characterised by distinct pathogen compositions, farm-specific distribution (Figure 4) and associated somatic cell counts (SCC) (Figure 5A). Profiles A, B, E and F included samples from all the farms. Farm 5 had no samples corresponding to profile C and only one corresponding to profile D, due to the near absence of the structuring pathogens for these profiles on this farm.

A full description of the pathogens present in each profile can be found in Additional file 4.

Profile A included most samples without pathogens (*n* = 252, i.e. 85%), and some with few pathogens (between 1 and 3 pathogens, *n* = 43, i.e. 15%). These samples had a median SCC of 14 (Interquartile range (IQR): 7–50) × 10^3^ cells/mL for the quarter of the udder that was analysed. Some samples exhibited very high SCC within this profile. Notably, 15.3% (*n* = 45) of the samples had SCC exceeding 200 × 10^3^ cells/mL. Among these, 19 samples contained *S. aureus*, 25 were pathogen-free, and one sample was associated with *E. coli*. Of these elevated-SCC samples, 82.2% (37 samples) originated from Farm 2.

Profile B comprised 250 samples, of which 202 (81%) of which contained at least one pathogen. NAS was the main pathogen structuring this group (*n* = 230, i.e. 92%). This profile also included 26 samples with *S. dysgalactiae* and 22 with *Enterococcus* spp. The median SCC for this profile was 27 (IQR: 12–253) × 10^3^ cells/mL.

Profile C contained 47 samples showing both *C. bovis* and *S. uberis*. Most of these also contained NAS (*n* = 36, i.e. 76.6%). *S. aureus* and yeasts were each present in nine samples. SCC of this group was the highest, with a median cell response of 931 (IQR: 239–1194) × 10^3^ cells/mL.

Profile D included 192 samples, all positive for *C. bovis*. Among these, some also contain NAS (*n* = 112, 58%). Less prevalent pathogens in this profile were yeasts (25%), *S. aureus* (22.4%), *S. dysgalactiae* (16.7%), *T. pyogenes*/*P. indolicus* (14.1%). These samples show the second highest median SCC of 93 (IQR: 27–695) × 10^3^ cells/mL, with 40% (*n* = 78) exhibiting SCC exceeding 200 × 10^3^ cells/mL. Among these 78 samples with elevated SCC, *S. aureus* was present in 30 samples, *S. dysgalactiae* in 20, and both pathogens in nine.

Profile E comprised 82 samples, all carrying *S. uberis*, with 55 also containing NAS (67%). The median SCC associated with these samples was 28 (IQR: 14–463) × 10^3^ cells/mL, with 37.8% of samples in this group showing a SCC greater than 200 × 10^3^ cells/mL.

Finally, profile F was characterised by the presence of NAS, *T. pyogenes/P. indolicus* and/or yeasts. NAS was detected in 76% (*n* = 108) of these samples. Among these samples, 30.3% contained only *T. pyogenes*/*P. indolicus*, 47.9% contained only yeasts and 21.8% contained both pathogens. In a 7-cluster partition, this pathogen profile splits according to the presence of yeast versus *T. pyogenes*/*P. indolicus*. These samples show a median SCC of 22 (IQR: 12–172) × 10^3^ cells/mL.

To assess the robustness of our clustering analysis, we repeated it after randomly removing 10%, 15%, and 20% of the samples (10 replicates per level; see Additional file 5). Of the 30-clustering analysis with partial data, 21 (i.e., 70%) led to clusters with the same characteristics, and an average of only 4.9% of samples were classified differently (with proportions of 2.6%, 5.1% and 6.7%, for the elimination of 10%, 15% and 20% of the data respectively). When clusters differed, the profile composed of NAS, yeasts and *T. pyogenes*/*P. indolicus* (profile F) was split into two clusters according to the presence of *T. pyogenes/P. indolicus* on the one hand and yeasts on the other. Most discrepancies between the initial analysis and replicates were associated with the merging of profiles differentiated by *S. uberis* presence in the initial analysis: profiles C and D, in 5 cases; profiles B and E, in 4 cases.

### SCC distribution across pathogens

SCC distribution varied across pathogens (Figure 5B). *S. aureus* was detected in 110 milk samples, with a median SCC of 744 (350—1440) × 10^3^ cells/mL. This species was present across all profiles defined by clustering, suggesting a specific impact on SCC. *S. dysgalactiae* was generally associated with SCC values above the mastitis threshold (200 × 10^3^ cells/mL), although some samples remained below this level. In contrast, *T. pyogenes/P. indolicus* and *Enterococcus* spp. were primarily associated with lower SCC values.

Other pathogens, including *S. agalactiae, Klebsiella* spp., *Mycoplasma* spp. and *Prototheca* spp., were detected in a limited number of samples. Specifically, *S. agalactiae* was identified in 10 samples (median SCC: 23.1 × 10^3^ cells/mL), *Klebsiella* spp. in 11 samples (16.2 × 10^3^ cells/mL), *Mycoplasma* spp. in 5 samples (16 × 10^3^ cells/mL), and *Prototheca* spp. in 11 samples (16 × 10^3^ cells/mL). Although generally associated with low SCC, these pathogens were too rare to contribute to the clustering-defined profiles.

For many pathogens, SCC distributions show a dichotomous pattern, with samples associated either low- or high-SCC values, with relatively few observations with intermediate values. This pattern was particularly evident for *S. dysgalactiae*, *S. uberis*, *C. bovis*, *E. coli* and yeasts. In the case of *S. uberis*, high SCC values were mostly observed in profile C, which also included *C. bovis*; conversely, lower SCC values were more frequently associated with profile E. Similarly, for *C. bovis*, samples with higher SCC were largely found in profile C, whereas those with low SCC associated to profile D.

NAS show a wide range of SCC values, with a low median, and a bimodal distribution (also apparent in profile B).

Overall pathogen-specific and profile-specific SCC distribution provided complementary insights (Figure 5). While certain pathogens such as *S. aureus* exhibited a consistent association with SCC values regardless of co-occurrence, others show SCC patterns that varied substantially depending on the microbial context. For instance, *S. uberis* exhibited a bimodal SCC distribution: low SCC were observed in profile E, whereas high SCC values were observed when *C. bovis* was also detected in profile C. These results suggest that SCC higher than 200 × 10^3^ cells/mL, indicating mastitis, can be associated to pathogen profiles, i.e. combinations of bacteria, rather than to individual pathogens alone.

### Relationship between milk profiles and bacterial abundance

**Figure 6:**
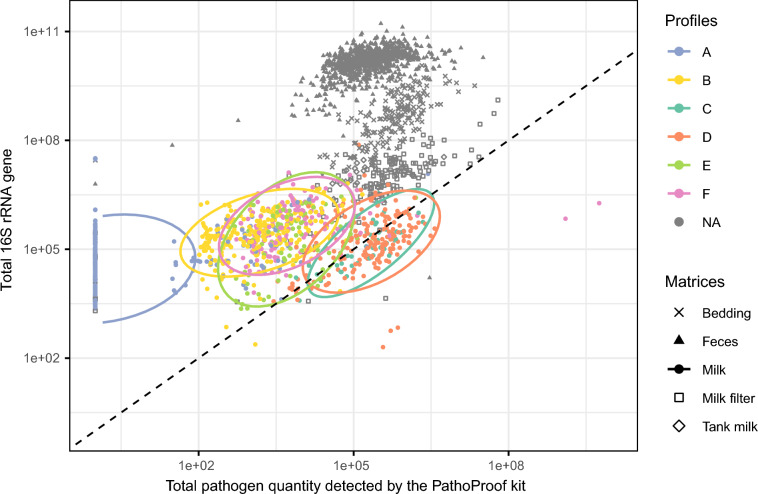
**Bacterial abundance** **(16S rRNA gene) depending on pathogen abundance (total copy number detected by PathoProof**^**™**^** kit for all pathogens) for each sample.** The shapes of the dots distinguish the matrices, while the colours of the dots reflect the profiles identified in the milk samples, with the legend showing the key pathogens defining each profile. The 95% confidence ellipses are drawn around the milk profiles, and the dotted line represents the y = x relationship.

To explore the contribution of detected pathogens to the overall bacterial community in each matrix, we compared total 16S rRNA gene copy numbers, as a proxy for total bacterial load, with the sum of the estimated pathogen copy numbers derived from data of the PathoProof^™^ panel. This comparison highlighted the occurrence of bacteria not detected by the qPCR detection test (Figure 6).

In milk samples, the relationship between total pathogen copy number and total bacterial abundances varied between profiles. Profiles C and D (*C. bovis* with variable presence of NAS and/or *S. uberis*) show a strong correspondence between pathogen abundance and the amount of 16S rRNA gene, suggesting that these bacteria are dominant. In contrast, profiles A and B, which were characterised by fewer pathogens, show larger quantities of 16S rRNA gene compared to pathogen abundance, suggesting the dominance of undetected non-pathogenic bacteria. Profiles E and F show an intermediate pattern, suggesting that other undetected bacteria were present. In comparison, samples of other matrices such as faeces, milk filter, and bedding, show similar total pathogen copy numbers, but revealed a much higher total bacterial abundance derived from 16S rRNA gene data, suggesting a substantial and larger number of non-pathogenic bacteria. For reference, milk filter, bedding and faeces show much larger quantities of 16S rRNA gene than milk samples.

Negative controls were included in all amplification runs to assess potential contamination. These controls show substantially lower amplification signals compared to biological samples, with average 16S rRNA gene concentrations being 352-fold lower in milk-related matrices and 1442-fold lower in faeces and bedding samples.

### Duration of infections by pathogens

**Figure 7:**
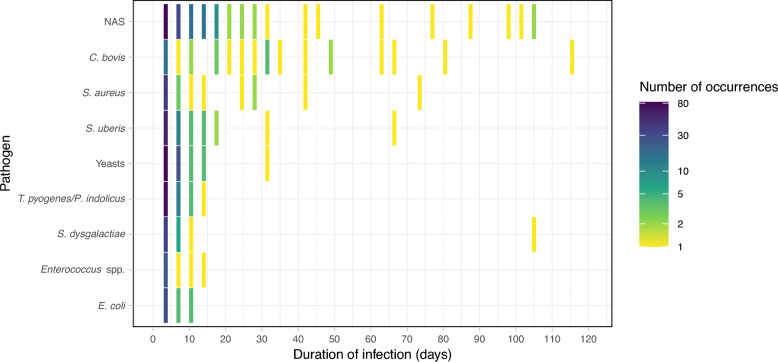
**Duration of infections caused by different pathogens, expressed in days**. Colours represent the number of infections per pathogen and duration. Sampling was performed every 3–4 days, so intervals between durations are approximately 3.5 days. An infection duration of 3.5 days corresponds to a single positive sample for the pathogen. NAS stands for non-*aureus* staphylococci. Pathogens detected in only one consecutive sample are not shown. Pathogens are ordered by the median infection duration.

Infection duration varied considerably among pathogens. *C. bovis* was involved in six infections lasting 60 days or more, suggesting persistent infections, and in twelve infections lasting between 20 and 45 days, making it the pathogen with the longest average infection duration. NAS followed, exhibiting a wide range of infection durations, from short episodes to prolonged cases lasting up to 126 days. *S. aureus* was responsible for two notably long infections of 80 and 63 days, alongside four shorter infections ranging from 10 to 31 days. *S. uberis* generally caused shorter infections but was also associated with two longer cases of 80 and 42 days. Yeasts, *Enterococcus* spp., *T. pyogenes/P. indolicus*, and *E. coli* were mostly involved in short-duration infections. *S. dysgalactiae* caused a prolonged infection lasting 122 days. These infection durations may be underestimated, as the sampling design did not always capture the full length of infection episodes, at the beginning or end of the observation period (Figure 7).

### Pathogen profile dynamics in milk samples

**Figure 8:**
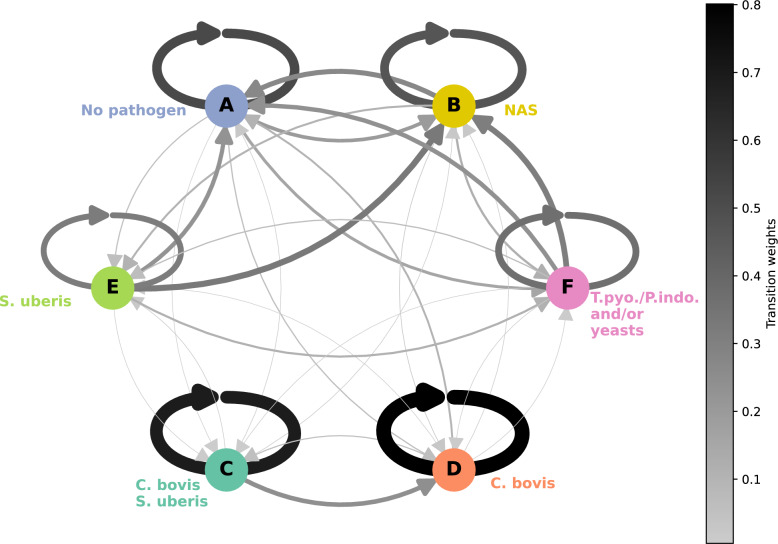
**Graph of profile transitions estimated by the Markov chain.** The nodes (circles) represent the profiles, while the arcs between the nodes illustrate the transition probabilities between these profiles. The width and colour of the arcs are proportional to the transition probabilities between profiles, with the colour scale ranging from grey (representing low probability) to black (representing high probability). Transitions reflect sample changes over 3- to 4-day intervals. The coloured names next to each node correspond to the pathogen structuring the associated profile. NAS = non-*aureus* staphylococci. T.pyo./P.indo. = *T. pyogenes/P. indolicus.*

To investigate further the temporal dynamics of pathogen in milk, we used a discrete-time Markov chain model [41], applied to both initial cluster-defined pathogen profiles or more stringent homogeneous infectious states (Additional file 7). Transition matrices were similar whether based on initial profiles or on the more stringent homogeneous states (see Additional file 8). Here, we present results based on the initial pathogen profiles (Figure 8).

For most profiles, the probability of remaining in the same profile, referred to as the persistence probability, was in general greater than the probability of transitioning to another profile (Figure 8). The highest persistence probabilities were observed for the profiles characterised by a high *C. bovis* frequency, with a probability of 0.68 for the C profile and 0.82 for the D profile, with and without *S. uberis* respectively (Additional file 8), suggesting persistent infections. For *C. bovis* and *S. uberis* (C) infections, samples came from 9 different cows, but 57% (*n* = 27) of these samples came from the same cow. All milk samples from this cow were associated with C status, except for two samples corresponding to E and D status. Thus, the probability of persistence of this state should be interpreted with caution. The persistence probability of profile A (pathogen-free profile) was 0.53, while the persistence probability of profile B (which includes only NAS) was 0.49. In contrast, profiles E (characterised by the presence of NAS and *S. uberis*) and F (NAS, *T. pyogenes/P. indolicus* and/or yeasts) had among the lowest persistence probabilities, with values of 0.34 and 0.36 respectively, indicating a short duration of these profiles. Furthermore, samples in profile E were more likely to transition to profile B with a probability of 0.37, than to remain in profile E, which had a persistence probability of 0.34.

Transitions were not random but rather followed structured patterns. Profiles E (NAS and *S. uberis*) and F (NAS, *T. pyogenes/P. indolicus* and yeasts), frequently transitioned towards profile A (pathogen free) or profile B (NAS only), with probabilities greater than 0.18. The presence of NAS in profile E was associated with transitions to profile B. In contrast, when NAS was absent, transitions tended to occur between E and A (Additional file 10). *T. pyogenes/P. indolicus* and/or yeasts were more likely to be lost than gained in the presence of NAS, with transitions from profile F to B more common than transitions from profile B to F. Similarly, losing NAS and moving to a pathogen-free profile, i.e. transition from profile B to profile A, was frequent (0.25).

Transitions into *C. bovis*-associated profiles were rare, e.g., 0.03 from profile B to profile D, and zero for transitions from profile E to profile C. The probability of losing *S. uberis* and moving from profile C to profile D was large (0.28). Within profile D (*C. bovis*), transitions between samples with and without NAS were frequent (Additional file 10).

The stationary distribution of the Markov chain model, representing long-term state occupancy, highlighted that the pathogen-free profile (A) and the profile with only NAS (B) would be the most frequent over time, with probabilities from the stationary distribution of 0.27 and 0.26 respectively (Additional file 8). Profile D was also frequent, with a probability from the stationary distribution of 0.20. Profile F had a lower probability from the stationary distribution of 0.14. Finally, profiles C and E, the two profiles containing *S. uberis*, were the least frequent, with probability from the stationary distribution of 0.05 and 0.09 respectively.

### Factors influencing profile dynamics

No trend was observed in the proportion of each milk profile, nor in the SCC, over the 4-month longitudinal follow-up period (Additional file 9).

**Figure 9:**
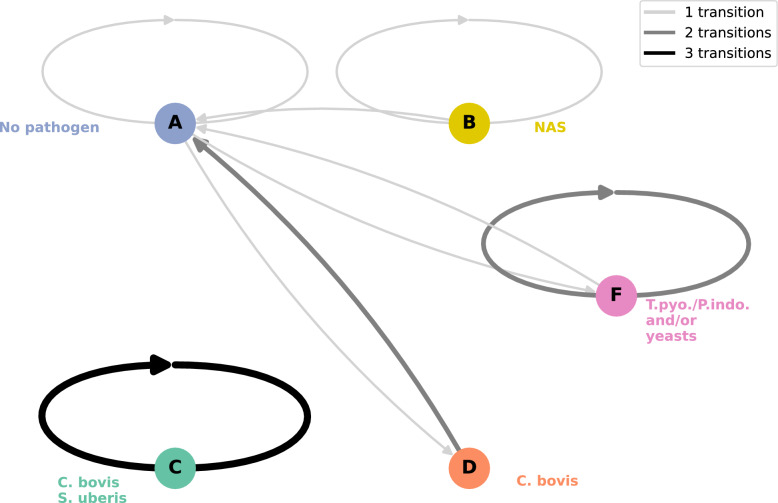
**Transitions between profiles following an antibiotic treatment**. The nodes (circles) represent the profiles, each arrow represents a change in profile (*n* = 13). The colours and thickness of the lines correspond to the associated transition numbers. The coloured names next to each node correspond to the pathogen structuring the associated profile. NAS = non-*aureus* staphylococci. T.pyo./P.indo. = *T. pyogenes/P. indolicus.*

During the sampling period, 14 antibiotic treatments were administered to nine cows, including seven intramammary treatments for mastitis (Additional file 6). Among the 459 observed transitions in pathogen profiles, only six occurred following an antibiotic treatment. Seven antibiotic treatments, including 4 intramammary, did not lead to any profile transition (Figure 9), while the remaining three were given either intramuscularly, intravenously, or intravaginally. One additional treatment was excluded as it occurred prior to the sampling period.

Three treatments were administered for *S. uberis*/*C. bovis* infections (profile C), none of which resulted in profile change. For profile F (NAS, *T. pyogenes*/P. indolicus and/or yeasts) two out of three treatments did not alter the profile, while one led to a transition to profile A. In profile D, the two treatments resulted in transition from profile D to A. When a treatment was applied to a cow in profile A (pathogen-free), the cow acquired pathogens following the treatment, resulting in a transition to profile D or F in two out of three cases, suggesting potential post-treatment colonization.

To explore whether transitions to profile A could result from an effective immune response, we examined changes in the SCC. The rate of SCC change (difference in SCC divided by the number of days between two samplings) was calculated and *t*-tests were conducted. When comparing the average rates of SCC change between the day of transition to profile A (*n* = 137) and the previous sampling date (−3.5 days) to other observed transitions (*n* = 322), no significant difference was found (*t*-test, t = −0.13, df = 384.90, *p*-value = 0.89). Additionally, somatic cell changes rates between the two sampling points before transition to profile A (samples −1 and −2, between 3.7 and 7 days before the profile change) was not significantly different either (t = 0.67, df = 169.25, *p*-value = 0.50). No consistent pattern in somatic cell change rates was observed when comparing transitions to profiles B, C, D, E or F with other profile changes, neither in the interval preceding a transition nor in the previous one (*t* tests: *p*-values > 0.05).

These results suggest that in the observed situations, neither antibiotic treatment nor SCC dynamics influenced the observed profile transitions in this study.

## Discussion

### Contribution and originality of the study design

This study provides an integrative view of mastitis pathogen associations and temporal dynamics, based on a longitudinal, multi-matrix sampling combined with qPCR pathogen detection and quantification, clustering of pathogens, and Markov chain modelling of transitions. By analysing in parallel milk, milk filters, faeces, bedding over a 4-month period in different individuals across six different farms and 2 years, we obtained a high-resolution description of infection dynamics.

To characterise pathogens colonizing the teat canal and potentially the udder, we adopted a foremilk sampling protocol targeting a single teat, favouring sensibility to detect the diversity of resident pathogens over diagnostic specificity toward the main mastitis causing pathogen. While this differs from standards in a clinical setting, like the National Mastitis Council protocol [36], and may increase contamination from microbes out of the udder, the structured and reproducible distribution of pathogens observed across cows, sessions and farms, along with non-random transitions between pathogen profiles, strongly suggest that our observations reflect biologically meaningful colonization dynamics rather than random contamination. Our study design may capture intramammary infections, but also the cumulative effects of repeated exposure to environmental and skin microbiota at the teat apex, which influence the presence and dynamics of mastitis pathogens.

### Sources of mastitis pathogen exposure on farms

Environmental pathogens such as *Trueperella pyogenes* and/or *Peptostreptococcus indolicus*, *Escherichia coli*, *Prototheca* spp., and yeasts were frequently detected in faeces and bedding. *Enterococcus* spp. are also pathogens mainly present in faeces, and their detection in 20% of bedding samples can therefore be explained by faecal contamination [42]. Non-*aureus* staphylococci (NAS) are present in all the compartments analysed. In fact, several studies have identified certain NAS species in bedding, on cow coats, nostrils, and teats [25, 43]. These organisms could therefore constitute a potential source of infection for the udder [44].

In contrast, some mastitis pathogens were found predominantly in milk-related compartments and were absent or rare in faeces or bedding, suggesting cow-to-cow transmission via direct contact or milking equipment. *Corynebacterium bovis* was present in most of the milk and milk filter samples but absent from faeces and bedding. Therefore, it can be assumed to be transmitted from one cow to another through direct contact between cows or via milking equipment [45]. This can be explained by its lipophilic [38] and microaerophilic nature [46] suggesting its presence in the mammary microbiota but scarcity in the environment [47]. In our study, *C. bovis* was absent from samples from one farm. This absence could be explained by the self-replacement of animals solely by internal births, thus limiting imports and perhaps preventing the introduction of *C. bovis*. It could also be due to the presence of other *Corynebacterium* species, not detected by the kit used, occupying its ecological niche, as previously reported [48, 49]. *Streptococcus uberis*, *Staphylococcus aureus* and *Streptococcus dysgalactiae* have similar distributions in the farm compartments, as shown by the principal component analysis. High copy numbers of these pathogens were found in the milk filter samples, in smaller proportions in the milk samples and occasionally in bedding, which could be explained by the contamination of bedding by milk. *S. aureus* is a contagious pathogen [45, 50, 51], while *S. uberis* and *S. dysgalactiae*, although mainly associated with environmental infections, can also be considered contagious pathogens [45, 52]. These pathogens, which are abundant in milk filter samples, seem to be transmitted between cows during the milking process.

### Different pathogen profiles within farms are also found across farms

A greater diversity of pathogens is present in the milk filters than in the individual milk samples, suggesting a heterogeneous distribution of pathogens within the herd. This supports the idea that cows harbour different individual pathogen profiles, which together contribute to the broader microbial diversity captured in the milk filters. Nevertheless, similar pathogen profiles were observed across farms, suggesting that microbial associations may reflect common infection patterns.

Despite exposure to shared sources of contamination, pathogen profiles remained heterogenous within farms, suggesting that cow level eco-epidemiological dynamics, including exposure, immune status, or microbial interactions, play a major role in shaping individual microbiota. Furthermore, transitions between pathogen profiles were structured and non-random, supporting the existence of recurrent pathogen assemblages in udder microbial communities.

### Persistence, prevalence, and ambiguous pathogenicity of non-*aureus* staphylococci in udder health

Not all 15 microbial taxa studied are consistently linked to high SCC. Among these taxa, NAS, a diverse group that includes many species considered as mastitis pathogens [53], were detected in 55% of our milk samples. NAS were particularly frequent in pathogen profile B, which represented the only profile defined solely by the presence of NAS and was the second most frequently observed pathogen profile across milk samples. NAS therefore appeared to play a dominant structuring role in this profile. In contrast, NAS was present in varying proportions in the other profiles (C, D, E, and F), so it does not have a structuring effect in these profiles, which could be explained by differences in species within the NAS group.

SCC values in NAS associated samples spanned a wide range. In profile B, in which other pathogens were rare, the median SCC was low, consistent with a healthy udder status based on SCC criteria [12], but SCC values show a wide distribution, and some samples were above the 200 × 10^3^ cells/mL threshold. This dispersion can be explained by several hypotheses. First, studies have reported that NAS can induce mastitis, but their direct effect on SCC seems to vary depending on the NAS species [47, 54, 55]. Second, elevated SCC can also correspond to cases where NAS co-occur with other pathogens, such as other *S. aureus* or *S. dysgalactiae.* The impact of host related variations, independent of infection by the detected pathogens, cannot be ruled-out.

Pathogen profile dynamics observed in this study revealed frequent transitions from profiles dominated by *S. uberis* (profile E) or by *T. pyogenes/P. indolicus* and/or yeasts (profile F) toward a NAS-dominated profile (profile B), and subsequently from a NAS-dominated profile to pathogen-free profile (A). This suggests that the NAS-dominated profile may represent an intermediate or transitional state of udder health. Furthermore, pathogen-free profile and NAS-dominated profile (A and B) were the most stable over time, supporting the hypothesis that following an infection, the mammary gland microbiota may either fully recover (profile A) or stabilise in a partially recovered, NAS-dominated profile (profile B). This model of partial or complete microbiota recovery is consistent with previous observations [32].

These findings raise questions about the classification of NAS as pathogens. Given their limited impact on SCC and association with stable pathogen profiles, it is plausible that some NAS species function as commensals of the udder rather than pathogens. However, due to the lack of species-level identification in this study, definitive conclusions cannot be drawn. This study quantified pathogen abundance from PathoProof^™^ PCR data instead of using a categorical approach, allowing detection of NAS, although limitations include challenges in the analysis of associations between NAS and *S. aureus* and in the detection of low concentrations.

Another potential limitation involves the presence of NAS on the teat apex or skin [56, 57], which may have influenced the composition of the teat canal studied here, despite rigorous disinfection protocols prior to sampling.

### Importance of co-infection in mastitis outcomes

Most mastitis studies focus on one or two specific pathogens, often overlooking the broader context of multi-pathogen associations. In contrast, our study simultaneously analysed the presence of 15 mastitis-associated bacterial taxa, revealing the significant role of co-infections in shaping mastitis outcomes.

Clustering analysis identified a co-infection pattern involving *Corynebacterium bovis* and *S. uberis* (profile C). This profile was associated with an elevated SCC, compared to profiles where these pathogens were detected alone (profiles D and E). In both profiles (D and E), the median SCC remained below the subclinical mastitis threshold. Notably, 76% of the high-SCC samples in *C. bovis-*associated profile (profile C) also contained major pathogens such as *S. aureus* or *S. dysgalactiae*, suggesting that although *C. bovis* may not induce a strong SCC alone it could facilitate co-infections with more virulent species [58].

Interestingly, *S. uberis* appears to have a different impact on SCC depending on whether *C. bovis* is also present. The much higher SCC in co-infected samples (profile C) compared to those with *S. uberis* alone (profile E) raises two main hypotheses: (i) a potential synergistic interaction between *C. bovis* and *S. uberis* that amplifies inflammation and SCC, or (ii) the presence of distinct *S. uberis* strains exhibiting different impacts on SCC. These findings align with studies suggesting strain-level variability in *S. uberis* pathogenicity [52, 59].

*S. uberis* infections can be short-term and effectively controlled by the host immune system, or persistent in some cases, with ongoing debate regarding the host and pathogen factors driving this dichotomy [52, 60]. In our study, most *S. uberis* infections were temporary; however, one cow with co-infection (*C. bovis* and *S. uberis*) exhibited a persistent infection throughout the sampling period, which contributed to the increased persistence of this infection state. Therefore, although it appears a promising hypothesis, further studies would be necessary to conclude on the impact of *C. bovis* on the prolonged persistence of *S. uberis*.

Clinical observations provide some support for these findings. In cows with co-infections with *S. uberis/C. bovis* (profile C), three antibiotic treatments were administered, but none resulted in a transition to a pathogen-free profile or a lower SCC profile. In contrast, two treatments applied to samples corresponding to the profile with *C. bovis* alone (profile D) led to a shift towards a pathogen-free profile (profile A). Although the number of treatments was limited and various antibiotics and administration routes were used, these observations suggest that co-infections may affect treatment success. Overall, these results highlight the importance of considering co-infections when evaluating the development of mastitis and its severity, as pathogen associations can significantly influence SCC and mastitis outcomes.

### Minor and major pathogens

Pathogens responsible for mastitis are generally classified into two groups, major and minor, based on their prevalence and their impact on symptom severity [10].

*S. aureus*, detected in 10.9% of milk samples, appeared across all pathogen profiles. This major mastitis pathogen induces a strong SCC [61, 62], often persisting over consecutive samplings but only present in a few cows. Indeed, *S. aureus* can cause chronic infections, particularly in its subclinical form [63, 64]. However, despite its clinical importance, *S. aureus* was not a structuring factor in the clustering of pathogen profiles in this study. This could be attributed to the low prevalence of *S. aureus* and the localised, persistent nature of its infections, which may reduce its influence on the clustering analysis.

Milk samples containing yeasts, *T. pyogenes/P. indolicus*, or a combination of these are grouped into pathogen profile F. This profile is also characterised by a low SCC, a high prevalence of NAS and the occurrence of yeasts in 17% of the milk sample studied; in milk, these likely belong to the *Candida* genus. Other studies found higher proportions of yeast in samples from udders with mastitis [65–67], but their impact on SCC depends on their co-occurrence with other pathogens [68, 69]. *T. pyogenes* and *P. indolicus* show a prevalence of 21.8% in samples from profile F. These anaerobic bacteria are recognised as major agents of summer mastitis [70, 71]. *T. pyogenes* is an opportunistic pathogen implicated in various infections (mastitis, abscesses, pneumonia, and metritis [72, 73]), whose prevalence increases with high temperatures and humidity [73]. Interpretation of this profile remains imprecise due to the diagnostic kit’s inability to differentiate yeast species or distinguish between *T. pyogenes* and *P. indolicus*; however, none of these agents appear to induce a strong SCC response during the winter season. If *T. pyogenes* is indeed present, as suggested, its limited impact in winter may reflect a shift from a pathogenic role in the summer to a more commensal-like presence in colder conditions.

Pathogen profiles dominated by *C. bovis* (profiles C and D) exhibited the highest persistence probabilities, indicating that cows infected with these profiles are likely to maintain them over time. *C. bovis*, considered as a minor pathogen [74] was detected in 23.7% of milk samples, making it the second most prevalent pathogen in this study. Moreover, it is associated with prolonged infection durations. Transitions from these profiles (C and D) to *C. bovis*-free profiles are rare, supporting the notion that *C. bovis* infections tend to be persistent [49]. This highlights the important role that minor pathogens can play, not only in shaping pathogen community profiles but also in influencing infection persistence.

### Factors influencing profile dynamics

Longitudinal observations revealed that direct transitions from a profile characterised by *S. uberis* (profile E) to a pathogen-free profile (profile A) or NAS-dominated profile occur (profile B) with no consistent priori increase in SCC nor any antimicrobial treatment. When NAS are present, transitions tend to favour the NAS-dominated profile (profile B), indicating a clearance of *S. uberis* while NAS persist. This suggests clearance of *S. uberis* that may result from microbial interactions acting specifically against *S. uberis*. This is consistent with findings showing that the mammary microbiota is capable of spontaneous recovery following infection [75].

Interestingly, both pathogen-free samples (profile A) and NAS-dominated samples (profile B) exhibit a substantial total bacterial load based on 16S rRNA gene quantification, exceeding the abundance of identified pathogens. This supports previous evidence suggesting the existence of a resident mammary microbiota, even in healthy quarters [21, 75, 76]. Transitions from a NAS-dominated profile (profile B) to pathogen-free profile (profile A) involve loss of NAS without a significant increase in SCC, suggesting that this shift may also be driven by microbial interactions within the microbiota rather than by SCC.

Clustering results indicate that co-occurrence of *S. uberis* and *C. bovis* (profile C) corresponds to subclinical mastitis, as indicated by elevated SCC. However, antibiotic treatments did not affect pathogen transitions for this profile, likely due to variation in efficacy [77] and low sample size. SCC changes also had no detectable effect, possibly because the sampling interval was too long to capture rapid immune responses [61]. Notably, samples from profiles containing *C. bovis* (profiles C and D) show bacterial loads comparable to total pathogen counts, suggesting that *C. bovis* and/or associated NAS form a substantial portion of the mammary microbiota. This observation is consistent with a study of human mastitis, which found that infections disrupted the microbiota, allowing opportunistic pathogens to become dominant [78]. This raises the hypothesis that a reduction in microbial diversity could impede recovery, as commensal bacteria potentially facilitate cure through interactions.

A limitation of this study lies in the focus on only 15 mastitis-associated pathogens. These pathogens vary in prevalence and impact, and it is likely that other bacteria not targeted by the diagnostic panel, (e.g., *Corynebacterium* spp., *Aerococcus* spp. [49, 79]) may contribute significantly to mastitis development and outcomes. Moreover, this targeted approach captures only a small fraction of the total bacterial population, overlooking potentially important structures and interactions within the commensal microbiota [21, 75, 76]. Studying the microbiota in these samples will provide a clearer understanding of the bacterial composition within the profiles and help identify non-pathogenic bacteria but potentially relevant bacteria.

## Conclusion

T﻿﻿his study highlights the multifactorial nature of mastitis, illustrating the different epidemiological dynamics of mastitis pathogens, their co-occurrence and impact on SCC. Our findings support the added-value of integrative, multi-pathogen longitudinal approaches to identify how co-infection dynamics complement information on mastitis obtained from a more transversal pathogen-based diagnostic. Pathogen associations changed over time and neglected pathogens such as *C. bovis* may play a central role in persistence and co-infection.

The recurrence of spontaneous transitions toward lower-SCC profiles questions the potential role for the native, non-pathogenic, udder microbiota in these systems, though contributions from the immune system cannot be ruled out. These diverse commensal bacteria may potentially facilitate cure through interactions, acting as microbial buffers or competitors. Moving beyond pathogen detection, future research could aim to identify microbial interactions that promote udder resilience and prevent persistent infection. Such insights could pave the way for more nuanced diagnostics and targeted interventions that reflect the ecological complexity of mastitis.

## Supplementary Information

**Additional file 1**. **Information on cow and farms.** Each sheet in the table corresponds to either cows or farms. Cow-specific data includes parity, breed, calving date, number of milk samples, and number of faecal samples. Farm-specific information includes the total number of cows and breeds, housing type, feeding practices, milk marketing, milking hygiene protocols, and the number of milk filter samples, bulk tank milk samples, and bedding samples.

**Additional file 2.** **Selection of the number of clusters.**

**Additional file 3.** **Representation of milk sample clustering results in multidimensional space for dimensions 1 and 2 (A), and for dimensions 3 and 4 (B). **The ellipses correspond to a 95% confidence interval for each cluster. Colours indicate the clusters identified by clustering. Percentages in brackets indicate the proportion of variance explained by each dimension.

**Additional file 4.** **Description of the pathogen composition of the different profiles.** Each sheet of the table corresponds to a profile.

**Additional file 5.** **Assessment of cluster robustness through random sample removal.**

**Additional file 6.** **Temporal dynamics of profiles for each cow based on clustering results. **Each row represents a cow, identified by the farm number and an individual letter (e.g., 3B). The x-axis shows the sampling timeline, expressed as the number of samples since the first day of sampling per period. Colours indicate to the profiles identified by clustering. Grey tiles ("NA") corresponds to time points where no sample was collected. Antibiotic treatments are categorised as follows: Intramammary: intramammary treatment for mastitis; Other: intramuscular or intravaginal treatment.

**Additional file 7.** **Definition of states.**

**Additional file 8.** **Transition between profiles and states.**

**Additional file 9.** **Evolution of profile proportions (A), and somatic cell counts (SCC) (B) over time (in days since the first sampling). **LOESS smoothing curves were fitted using the geom_smooth function for both sampling periods.

**Additional file 10.** **Impact of NAS on profiles transitions.**

## Data Availability

The dataset and analysis scripts supporting the conclusions of this article are available in the INRAE Data repository: Lirot Hélène, Bompard Anaïs, 2025, “Mastitis pathogen and 16S qPCR results and associated metadata collected in Auvergne farms (France)” [80].
