# Supplementary material for: Longitudinal analysis reveals transitions in pathogen profiles associated with mastitis in dairy cows
Source: Vet Res. 2025 Dec 18;56:231. doi: 10.1186/s13567-025-01665-y (PMC12715916; doi:10.1186/s13567-025-01665-y)
Supplement: Supplementary file 2 — Additional file 2. Selection of the number of clusters. [file 13567_2025_1665_MOESM2_ESM.docx]

**Additional file 2: Selection of the number of clusters**

To identify the main associations of pathogens in the milk samples, a clustering analysis was carried out. Several approaches were used to determine the optimum number of clusters (Figure 1). The elbow in the within-cluster sum of squares (WSS) curve (Figure 1A) and the Silhouette (1B) both indicate that two clusters are a relevant choice, while the gap statistic method (Figure 1C) suggests a group number of 12, but this number increases when the group limit for calculating this statistic is raised.


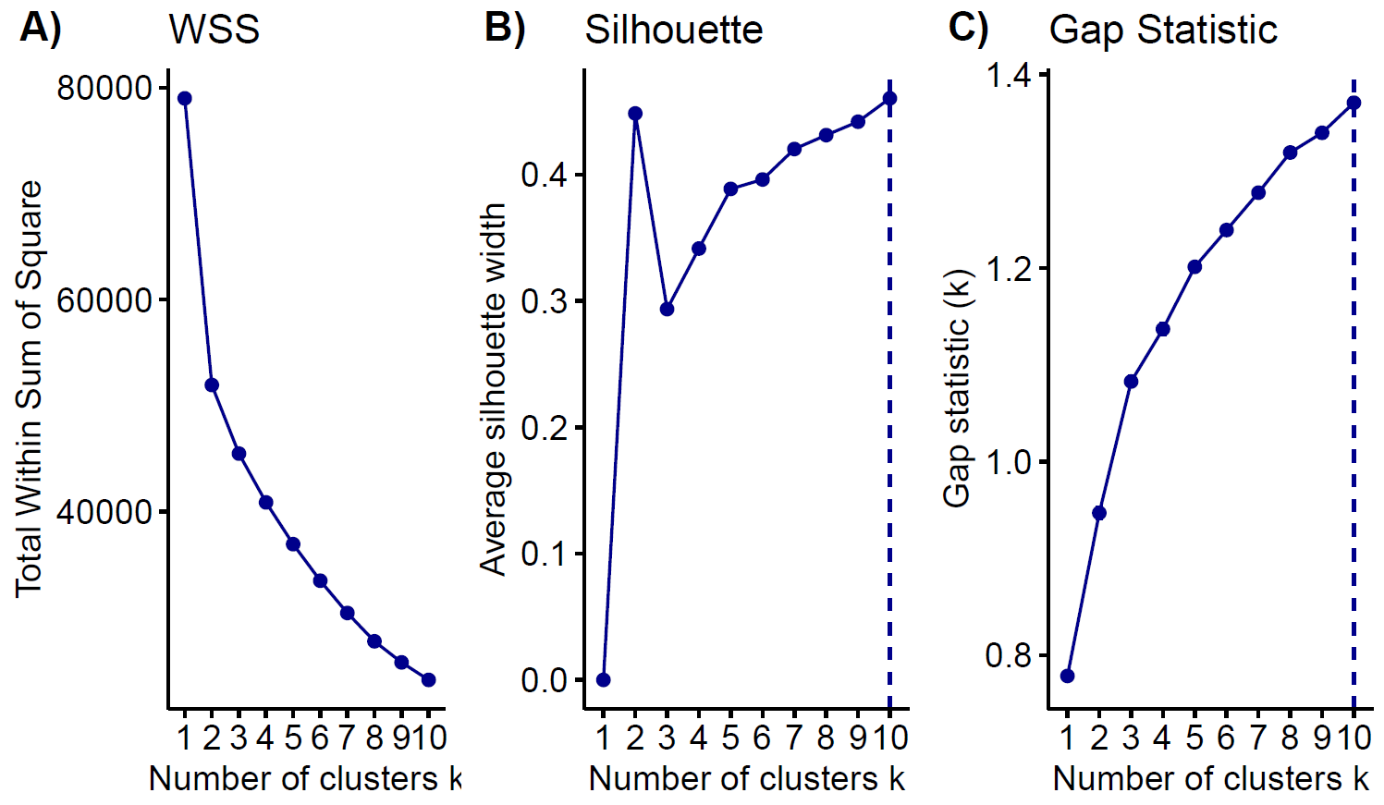


**Figure 1: Criteria for selecting the optimum number of clusters for clustering:** A) within-cluster sum of squares curve, B) Silhouette index, and C) gap statistic method.

However, partitioning into only 2 clusters was not sufficient to capture the diversity of milk pathogen profiles and masked biologically relevant infection scenarios. In fact, there are different types of mastitis with different infection patterns, and the grouping structure into 2 clusters did not allow us to achieve this level of detail. Thus, clustering analyses were carried out for different numbers of clusters, in order to assess the impact of this variation on the observed profiles and to validate the number of clusters to be chosen for further analysis.

Cluster characteristics revealed that the initial separation into 2 clusters is mainly influenced by the presence of *C. bovis*. As the number of clusters increased, the groups split progressively according to the presence of other key pathogens such as NAS, *T. pyogenes/P. indolicus, S. uberi*s and yeasts.

Cluster characteristics according to the number of groups (Figure 2):

- 2 clusters: Separation is mainly influenced by the presence of *C. bovis*.
- 3 clusters: The group without *C. bovis* is divided according to the presence of NAS.
- 4 clusters: The newly formed group is split into two, according to the presence of *T.* pyogenes*/P. indolicus* and yeasts.
- 5 clusters: The group consisting solely of NAS is divided according to the presence or absence of *S. uberis*.
- 6 clusters: The group with *C. bovis* is subdivided according to the presence of *S. uberis*.
- 7 clusters: The group containing NAS, yeasts and *T. pyogenes*/*P. indolicus* is split into two: one subgroup with yeasts, and another with *T. pyogenes*/*P. indolicus*.
- 8 clusters: The group containing *C. bovis* without *S. uberis* is divided according to the presence of *S. dysgalactiae* or yeasts versus the absence of these pathogens.
- 9 clusters: The group previously formed was in turn split to separate *S. dysgalactiae* and yeasts.


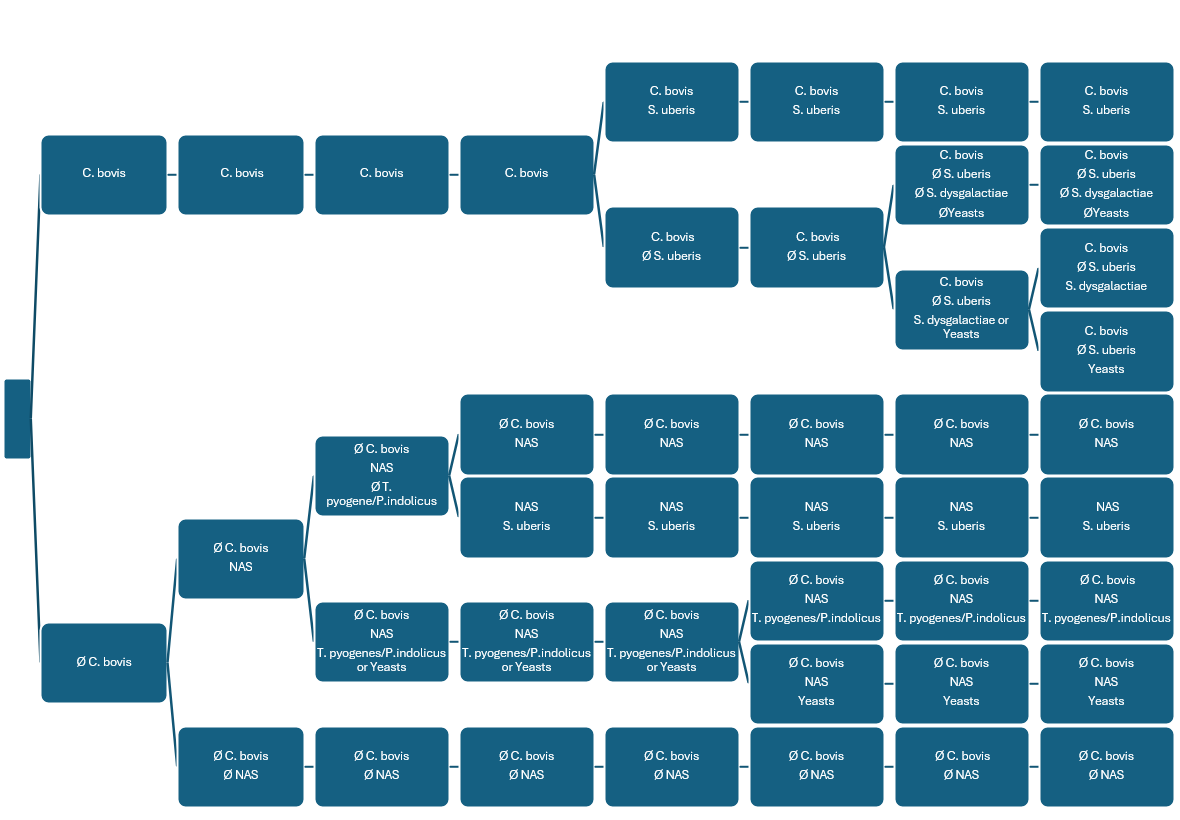


**Figure 2: Cluster characteristics according to the number of groups**

After evaluation, a partitioning into 6 clusters was retained as an optimal compromise. This choice provides biological relevance while maintaining sufficient diversity to analyse pathogen profiles. This configuration highlights the distribution of *S. uberis*, a major mastitis pathogen. This number of clusters provides an optimal balance between the granularity of the groups and their coherence, avoiding excessive fragmentation or structuring that is too influenced by individuals.

The study used Ward's clustering method despite its sensitivity to outliers to identify general pathogen patterns in milk samples, ensuring that clusters represented multiple cows, and the number of clusters was chosen as a compromise based on data characteristics and sampling diversity.
