# Supplementary figures and images for: Longitudinal analysis reveals transitions in pathogen profiles associated with mastitis in dairy cows
Source: Vet Res. 2025 Dec 18;56:231. doi: 10.1186/s13567-025-01665-y (PMC12715916; doi:10.1186/s13567-025-01665-y)

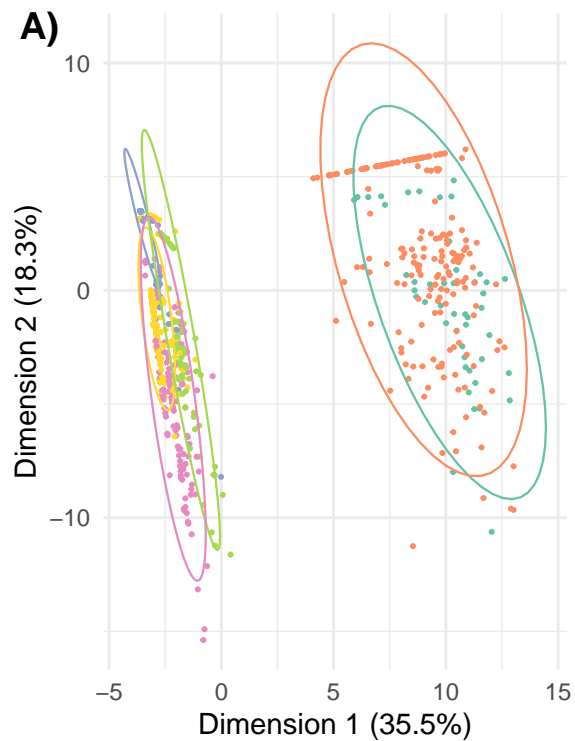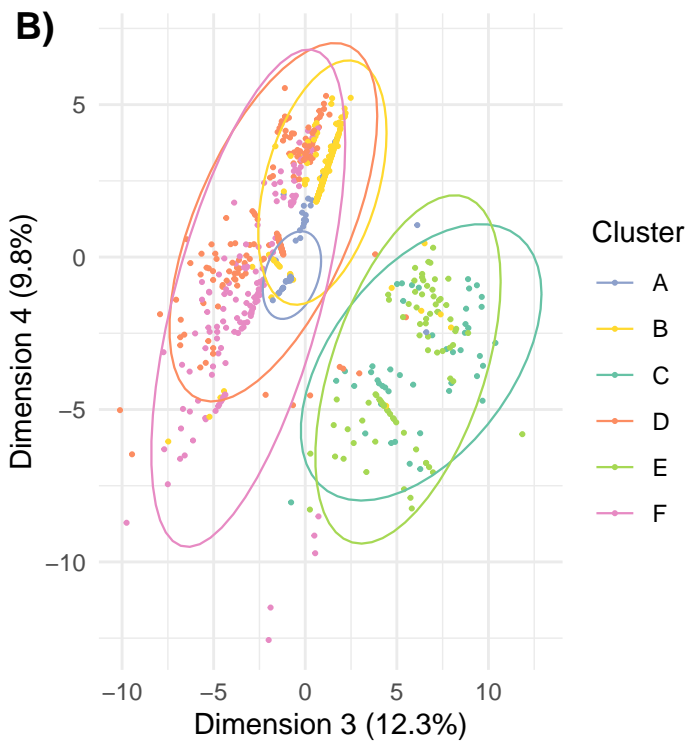

Supplement: Supplementary file 3 — Additional file 3. Representation of milk sample clustering results in multidimensional space for dimensions 1 and 2 (A), and for dimensions 3 and 4 (B). The ellipses correspond to a 95% confidence interval for each cluster. Colours indicate the clusters identified by clustering. Percentages in brackets indicate the proportion of variance explained by each dimension. [file 13567_2025_1665_MOESM3_ESM.pdf]

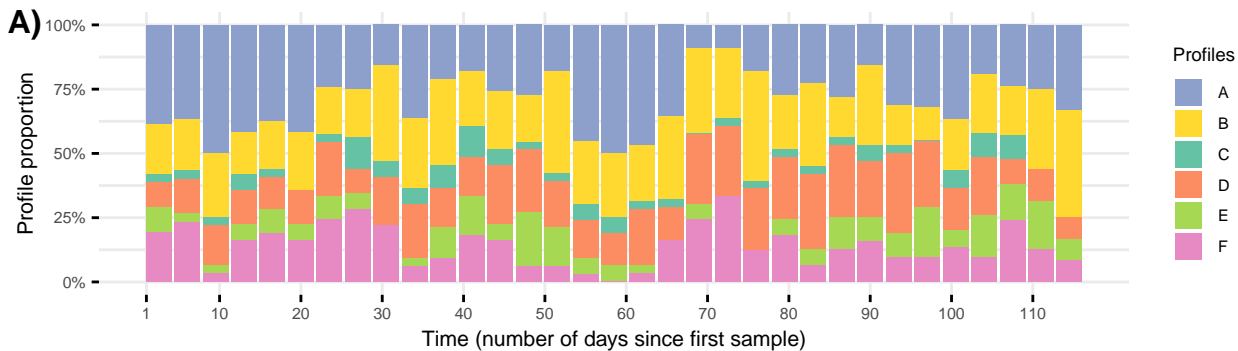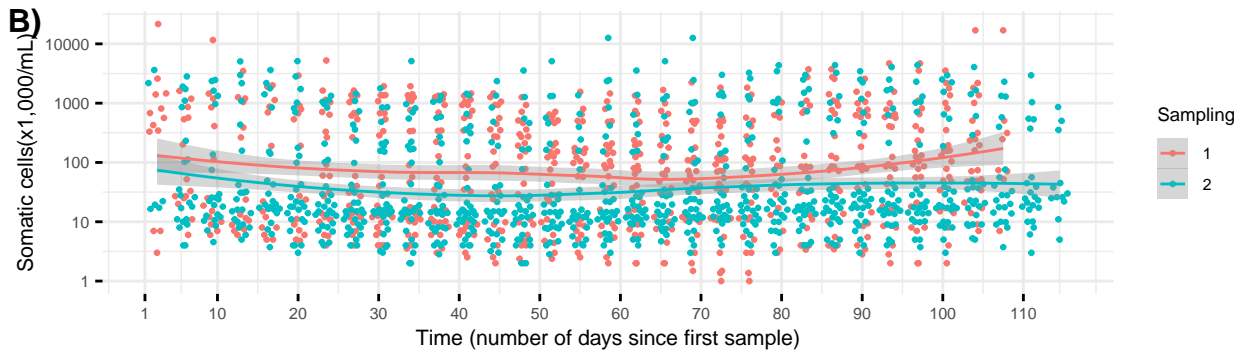

Supplement: Supplementary file 9 — Additional file 9. Evolution of profile proportions (A), and somatic cell counts (SCC) (B) over time (in days since the first sampling). LOESS smoothing curves were fitted using the geom_smooth function for both sampling periods. [file 13567_2025_1665_MOESM9_ESM.pdf]
