# Supplementary material for: Longitudinal analysis reveals transitions in pathogen profiles associated with mastitis in dairy cows
Source: Vet Res. 2025 Dec 18;56:231. doi: 10.1186/s13567-025-01665-y (PMC12715916; doi:10.1186/s13567-025-01665-y)
Supplement: Supplementary file 6 — Additional file 6. Temporal dynamics of profiles for each cow based on clustering results. Each row represents a cow, identified by the farm number and an individual letter (e.g., 3B). The x-axis shows the sampling timeline, expressed as the number of samples since the first day of sampling per period. Colours indicate to the profiles identified by clustering. Grey tiles ("NA") corresponds to time points where no sample was collected. Antibiotic treatments are categorised as follows: Intramammary: intramammary treatment for mastitis; Other: intramuscular or intravaginal treatment. [file 13567_2025_1665_MOESM6_ESM.pdf]

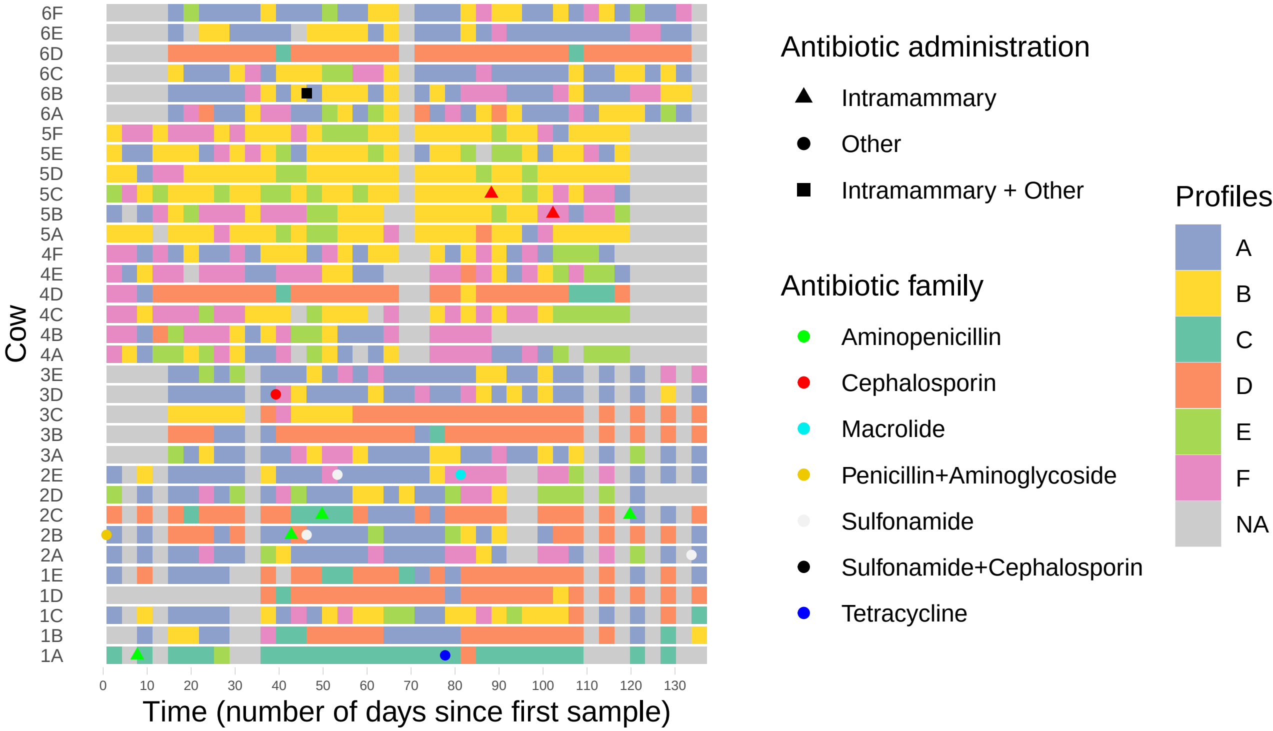

Cow
6F
6E
6D
6C
6B
6A
5F
5E
5D
5C
5B
5A
4F
4E
4D
4C
4B
4A
3E
3D
3C
3B
3A
2E
2D
2C
2B
2A
1E
1D
1C
1B
1A
0 10 20 30 40 50 60 70 80 90 100 110 120 130
Time (number of days since first sample)
Antibiotic administration
Intramammary
Other
Intramammary + Other
Antibiotic family
Aminopenicillin
Cephalosporin
Macrolide
Penicillin+Aminoglycoside
Sulfonamide
Sulfonamide+Cephalosporin
Tetracycline
Profiles
A
B
C
D
E
F
NA
