# Supplementary material for: Longitudinal analysis reveals transitions in pathogen profiles associated with mastitis in dairy cows
Source: Vet Res. 2025 Dec 18;56:231. doi: 10.1186/s13567-025-01665-y (PMC12715916; doi:10.1186/s13567-025-01665-y)
Supplement: Supplementary file 7 — Additional file 7. Definition of states. [file 13567_2025_1665_MOESM7_ESM.docx]

**Additional file 7: Definition of states**

Based on the description of the profiles resulting from the clustering, the defining characteristics of each state can be identified:

- A*: Absence of the 15 pathogens.
- B*: Presence of NAS and absence of *C. bovis, S. uberis*, yeasts and *T. pyogenes*/*P. indolicus*.
- C*: Presence of *C. bovis* and *S. uberis*.
- D*: Presence of *C. bovis* and absence of *S. uberis*.
- E*: Presence of *S. uberis* and absence of *C. bovis*.
- F*: Presence of *T. pyogenes*/*P. indolicus* and/or yeasts, and absence of *C. bovis* and *S. uberis.*
- Unclassifiable: All samples not allocated in any of the previous states.

**Table 2 -** **Definition of states for the Markov model.**

| States | Definition | R pseudo code# | Sample size |
| --- | --- | --- | --- |
| A* | Absence of the 15 pathogens | *C_bovis == 0 & E_coli ==0 & Entero_sp == 0 & Klebsie_sp == 0 & M_bovis == 0 & M_sp == 0 & NAS == 0 & Proto_sp == 0 & S_aureus == 0 & S_agala == 0 & S_dysgala == 0 & S_uberis == 0 & Yeasts == 0 & T_pyo_P_indo == 0* | 252 |
| B* | Presence of NAS and absence of *C. bovis, S. uberis*, yeasts and *T. pyogenes*/*P. indolicus* | *NAS > 0 & C_bovis == 0 & Str_uberis == 0 & Yeasts == 0 & T_pyo_P_indo == 0* | 238 |
| C* | Presence of *C. bovis* and *S. uberis* | *C_bovis > 0 & Str_uberis > 0* | 55 |
| D* | Presence of *C. bovis* and absence of *S. uberis* | *C_bovis > 0 & Str_uberis == 0* | 184 |
| E* | Presence of *S. uberis* and absence of *C. bovis* | *Str_uberis > 0 & C_bovis == 0* | 95 |
| F* | Presence of *T. pyogenes*/*P. indolicus* and/or yeasts, and absence of *C. bovis* and *S. uberis* | *(Yeasts > 0 \| T_pyo_P_indo > 0) & C_bovis == 0 & Str_uberis == 0* | 145 |


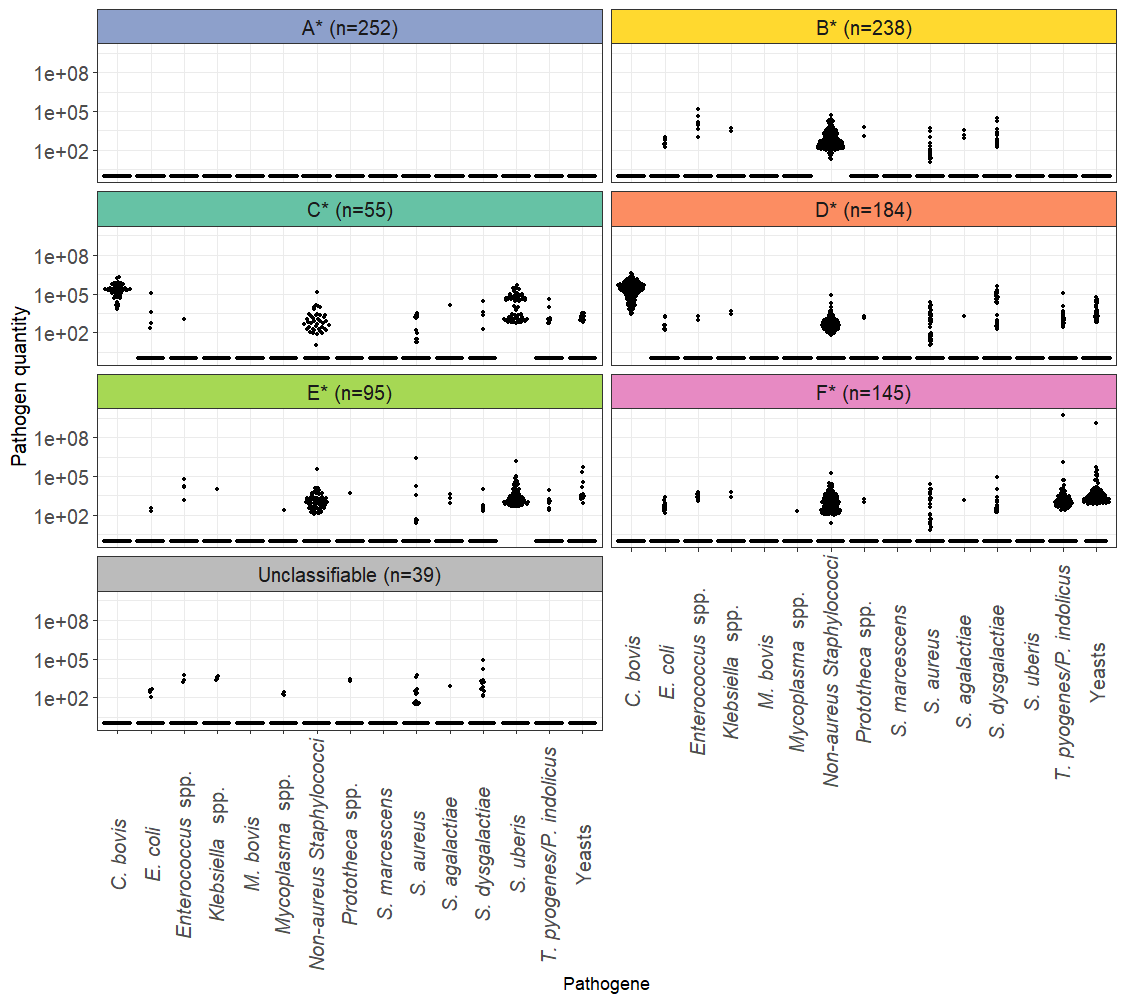


**Figure 1: Distribution of pathogen quantities for each state.** The x-axis (log-transformed) indicates the quantity of pathogens detected, and the y-axis shows the individual pathogens. Results are separated by states. “N” corresponds to the number of samples per state.

It is possible to reassign a state to each sample (Figure 1), using the previous characteristics. As a result, 84 samples were found to be unclassifiable or assigned to a different group (Table 1). The clustering misclassification rate is thus estimated at 8.33%. 39 samples could not be classified with a state.

**Table 1: Confusion matrix between clustering profiles and states.** The numbers correspond to the number of samples. The letters with a star correspond to the states (A* to F*). The numbers in bold correspond to the number of samples classified in the correct profile.

|  | | **Corrected profiles / States** | | | | | | |
| --- | --- | --- | --- | --- | --- | --- | --- | --- |
|  |  | **A*** | **B*** | **C*** | **D*** | **E*** | **F*** | **Unclassifiable** |
| **Clustering profiles** | **A** | **252** | 16 | 0 | 0 | 2 | 1 | 24 |
|  | **B** | 0 | **222** | 0 | 0 | 6 | 7 | 15 |
|  | **C** | 0 | 0 | **47** | 0 | 0 | 0 | 0 |
|  | **D** | 0 | 0 | 8 | **184** | 0 | 0 | 0 |
|  | **E** | 0 | 0 | 0 | 0 | **82** | 0 | 0 |
|  | **F** | 0 | 0 | 0 | 0 | 5 | **137** | 0 |

Among the unclassified samples (n=39), there is a majority with a single pathogen: *E. coli* (n=5), *Enterococcus* spp. (n=3), *Klebsiella* spp. (n=3), *Mycoplasma* spp. (n=3), *Prototheca* spp. (n=2), *S. aureus* (n=10), *S. agalactiae* (n=1) and *S. dysgalactiae* (n=9), as well as three samples containing two pathogens. We hypothesize that some of these pathogens may play a structuring role and that the samples may be divided into states. It is also possible that some of these pathogens are not structuring, however, it is not possible to test this hypothesis with this dataset, as we cannot distinguish between the lack of statistical power due to the small number of samples containing these pathogens and the real absence of a structuring effect. A study involving samples from more farms and cows would allow us to establish and define the potential other states of the cows' udders based on their composition. Studying the microbial diversity associated with these samples would also enable us to determine whether they can be considered as one or several states.
