# Supplementary material for: Longitudinal analysis reveals transitions in pathogen profiles associated with mastitis in dairy cows
Source: Vet Res. 2025 Dec 18;56:231. doi: 10.1186/s13567-025-01665-y (PMC12715916; doi:10.1186/s13567-025-01665-y)
Supplement: Supplementary file 8 — Additional file 8. Transition between profiles and states. [file 13567_2025_1665_MOESM8_ESM.docx]

**Additional file 8: Transition between profiles and states**

- **Clustering results: profiles**

**Table 1: Transition matrix of profiles estimated by the Markov chain.** The rows represent the initial profile, while the columns correspond to the destination profile.

|  | **A** | **B** | **C** | **D** | **E** | **F** |
| --- | --- | --- | --- | --- | --- | --- |
| **A** | 0.528 | 0.198 | 0.004 | 0.048 | 0.060 | 0.161 |
| **B** | 0.248 | 0.487 | 0.000 | 0.031 | 0.111 | 0.124 |
| **C** | 0.025 | 0.000 | 0.675 | 0.275 | 0.025 | 0.000 |
| **D** | 0.072 | 0.026 | 0.065 | 0.817 | 0.007 | 0.013 |
| **E** | 0.183 | 0.366 | 0.000 | 0.000 | 0.338 | 0.113 |
| **F** | 0.242 | 0.311 | 0.008 | 0.015 | 0.061 | 0.364 |

**Table 2: Probability from the stationary distribution for each profile estimated by the Markov chain.**

|  | **A** | **B** | **C** | **D** | **E** | **F** |
| --- | --- | --- | --- | --- | --- | --- |
| **Stationary probability** | 0.273 | 0.260 | 0.046 | 0.197 | 0.085 | 0.139 |

- **After correction of pathogen profiles: states**

**Table 3: Transition matrix of states (without unclassifiable samples) estimated by the Markov chain.** The rows represent the initial state, while the columns correspond to the destination state.

|  | **A*** | **B*** | **C*** | **D*** | **E*** | **F*** |
| --- | --- | --- | --- | --- | --- | --- |
| **A*** | 0.505 | 0.184 | 0.015 | 0.044 | 0.068 | 0.184 |
| **B*** | 0.203 | 0.485 | 0.000 | 0.030 | 0.139 | 0.144 |
| **C*** | 0.022 | 0.000 | 0.674 | 0.283 | 0.022 | 0.000 |
| **D*** | 0.068 | 0.027 | 0.075 | 0.808 | 0.007 | 0.014 |
| **E*** | 0.177 | 0.354 | 0.000 | 0.000 | 0.354 | 0.114 |
| **F*** | 0.235 | 0.318 | 0.008 | 0.015 | 0.083 | 0.341 |

**Table 4: Probability from the stationary distribution for each state estimated by the Markov chain (without unclassifiable samples).**

|  | **A*** | **B*** | **C*** | **D*** | **E*** | **F*** |
| --- | --- | --- | --- | --- | --- | --- |
| **Stationary probability** | 0.241 | 0.258 | 0.059 | 0.193 | 0.104 | 0.146 |

- **Comparison of transition matrices between pathogen profiles and states**


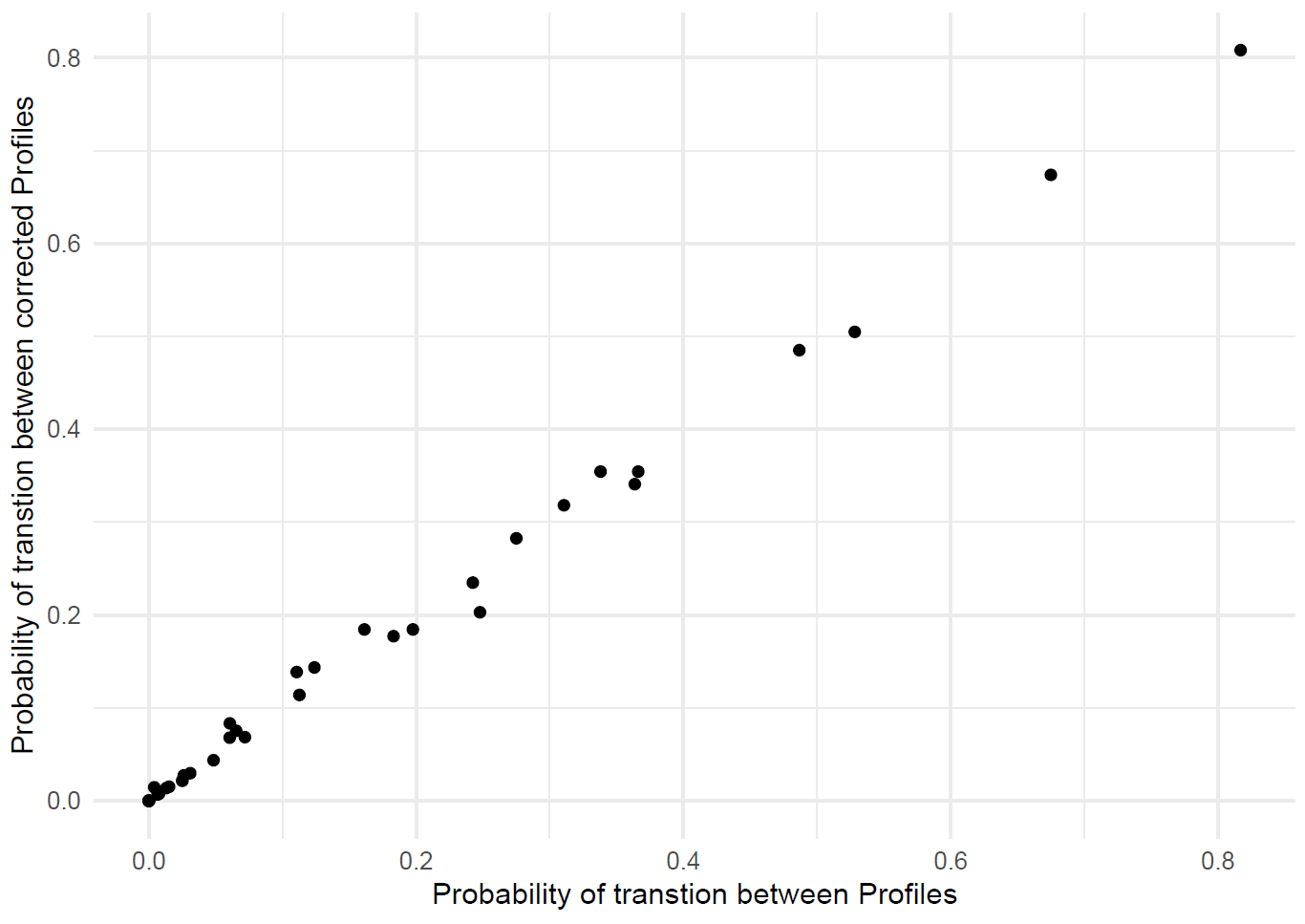


**Figure 1: Comparison of transition probabilities.** The x-axis represents transition probabilities for the clustering results, while the y-axis shows the transition probabilities for the states. The points correspond to the different transitions (e.g., transition from A to B and from A* to B*)
