# Supplementary material for: Longitudinal analysis reveals transitions in pathogen profiles associated with mastitis in dairy cows
Source: Vet Res. 2025 Dec 18;56:231. doi: 10.1186/s13567-025-01665-y (PMC12715916; doi:10.1186/s13567-025-01665-y)
Supplement: Supplementary file 10 — Additional file 10. Impact of NAS on profiles transitions. [file 13567_2025_1665_MOESM10_ESM.docx]

**Additional file 9: Impact of NAS on profiles transitions**

To assess the impact of NAS (non-*aureus s*taphylococci) on profile transitions, we split profiles C to F based on the presence or absence of NAS. This resulted in a total of 10 profiles, with the following sample counts:

| Profiles | A | B | C | C | D | D | E | E | F | F |
| --- | --- | --- | --- | --- | --- | --- | --- | --- | --- | --- |
| Presence of NAS |  |  | No | Yes | No | Yes | No | Yes | No | Yes |
| Number of samples | 295 | 250 | 36 | 11 | 112 | 80 | 55 | 27 | 108 | 34 |


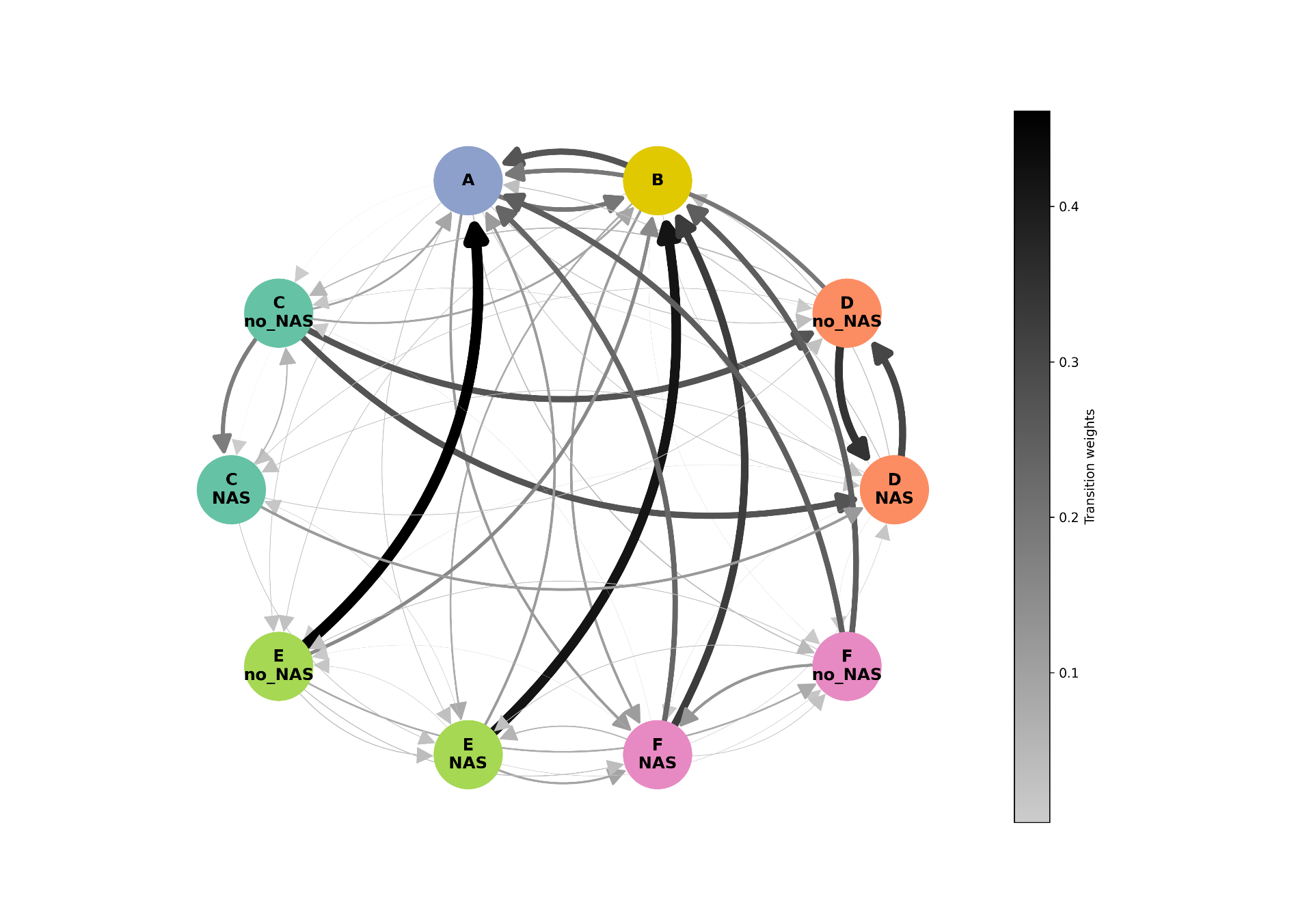


**Figure 1: Graph of profile transitions based on the presence or absence of NAS.** The nodes (circles) represent the profiles, while the arcs between nodes illustrate the transition probabilities between profiles. The width and colour intensity of the arcs are proportional to the transition probabilities, with a colour scale ranging from grey (indicating low probability) to black (indicating high probability). Transitions reflect changes observed over 3-to-4-day sampling intervals.

**Table 1: Transition matrix of profiles based on the presence or absence of NAS estimated by the Markov chain.** The rows represent the profile, while the columns correspond to the destination profile.

|  | **A** | **B** | **C**  **NAS** | **C**  **no_NAS** | **D**  **NAS** | **D**  **no_NAS** | **E**  **NAS** | **E**  **no_NAS** | **F**  **NAS** | **F**  **no_NAS** |
| --- | --- | --- | --- | --- | --- | --- | --- | --- | --- | --- |
| **A** | 0.514 | 0.195 | 0.004 | 0.004 | 0.028 | 0.035 | 0.032 | 0.028 | 0.113 | 0.046 |
| **B** | 0.270 | 0.471 | 0.000 | 0.000 | 0.025 | 0.008 | 0.078 | 0.029 | 0.111 | 0.008 |
| **C**  **NAS** | 0.000 | 0.000 | 0.765 | 0.059 | 0.118 | 0.029 | 0.029 | 0.000 | 0.000 | 0.000 |
| **C**  **no_NAS** | 0.091 | 0.091 | 0.182 | 0.091 | 0.273 | 0.273 | 0.000 | 0.000 | 0.000 | 0.000 |
| **D**  **NAS** | 0.037 | 0.037 | 0.028 | 0.019 | 0.546 | 0.306 | 0.000 | 0.009 | 0.009 | 0.009 |
| **D**  **no_NAS** | 0.192 | 0.000 | 0.026 | 0.051 | 0.346 | 0.385 | 0.000 | 0.000 | 0.000 | 0.000 |
| **E**  **NAS** | 0.113 | 0.415 | 0.019 | 0.000 | 0.000 | 0.000 | 0.321 | 0.019 | 0.094 | 0.019 |
| **E**  **no_NAS** | 0.462 | 0.154 | 0.000 | 0.000 | 0.000 | 0.000 | 0.038 | 0.231 | 0.038 | 0.077 |
| **F**  **NAS** | 0.234 | 0.327 | 0.000 | 0.009 | 0.019 | 0.000 | 0.056 | 0.009 | 0.318 | 0.028 |
| **F**  **no_NAS** | 0.250 | 0.250 | 0.000 | 0.000 | 0.000 | 0.000 | 0.031 | 0.031 | 0.125 | 0.313 |

**Table 2: Probability from the stationary distribution for each profile based on the presence or absence of NAS.**

|  | ***A*** | ***B*** | ***C***  ***NAS*** | ***C***  ***no_NAS*** | ***D***  ***NAS*** | ***D***  ***no_NAS*** | ***E***  ***NAS*** | ***E***  ***no_NAS*** | ***F***  ***NAS*** | ***F***  ***no_NAS*** |
| --- | --- | --- | --- | --- | --- | --- | --- | --- | --- | --- |
| **Stationary probability** | 0.284 | 0.244 | 0.041 | 0.012 | 0.119 | 0.086 | 0.054 | 0.025 | 0.103 | 0.032 |
